# Optimizing Surface Wettability for Confined H_2_–CH_4_ Clathrates in Porous Activated Carbon

**DOI:** 10.1021/acsami.5c18795

**Published:** 2026-01-14

**Authors:** Erling Velten Rothmund, Jianying He, Zhiliang Zhang, Senbo Xiao

**Affiliations:** Department of Structural Engineering, 8018 Norwegian University of Science and Technology (NTNU), Trondheim 7491, Norway

**Keywords:** activated carbon, hydrogen storage, gas clathrate hydrates, wettability, porous media, methane

## Abstract

Hydrogen (H_2_) clathrate hydrates are emerging solid-state media for safe and efficient hydrogen storage, yet practical deployment is hindered by slow formation kinetics and limited storage capacities under mild conditions. Confinement within nanoporous media, particularly activated carbons, substantially alleviates these limitations, but the governing role of interfacial chemistry remains unclear. Here, molecular dynamics simulations identify a predictive wettability window that maximizes binary H_2_–CH_4_ clathrate formation, stability, and gas uptake in nanoporous carbons, with optimal performance at moderate hydrophilicity (water contact angle ≈ 43°). This optimum arises from a balance between excessive interfacial-water ordering at strongly hydrophilic surfaces and gas–water phase separation at strongly hydrophobic surfaces. At this wettability, the critical pore size required for stable enclathration is minimized, expanding the clathrate-accessible pore volume and enabling higher gas storage capacity. Furthermore, a dual-storage mechanism in hierarchical porous media is demonstrated across a broad range of surface chemistries, integrating micropore physisorption with meso/macropore enclathration to significantly enhance gas storage capacity. These findings yield experimentally testable material design rules that connect surface wettability and porosity to gas storage performance. Because wettability and porosity are tunable via surface functionalization and synthesis conditions, these rules directly inform the design of porous carbons and related materials for hydrogen and methane storage technologies.

## Graphical abstract

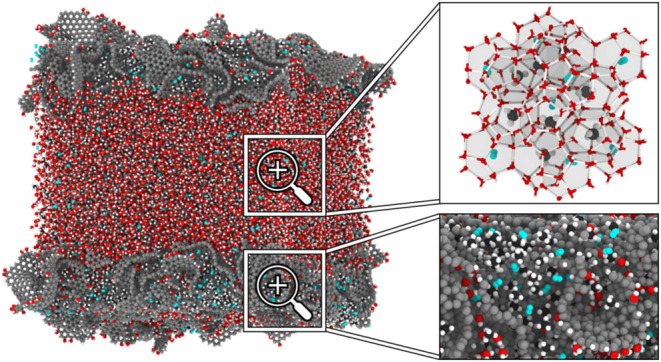

## 1 Introduction

Molecular hydrogen (H_2_) is a promising green fuel, particularly for decarbonizing hard-to-abate sectors,^1,2^ but its low volumetric density poses challenges for safe and efficient storage and transport. Gas clathrate hydrates are crystalline, water-based solids capable of trapping (*enclathrating*) gas molecules within nanoscale cages,^3,4^ which have emerged as a promising medium for H_2_ storage.^5–7^ Contrary to gaseous H_2_, H_2_-clathrate is nonexplosive and metastable at ambient pressures.^8–11^ Contrary to liquid H_2_, which requires cryogenic temperatures, H_2_-clathrate forms at mild-temperature conditions close to −10 °C, depending on pressure.^12^ In addition to purely consisting of water and gas, these properties make gas clathrates attractive candidates for sustainable H_2_ storage.

The greatest challenge in clathrate-based gas storage is the initial formation, which typically requires high pressure and long formation times.^8^ In contrast, fully formed clathrates can persist metastably under much milder conditions.^9–11,13^ Porous host media have been shown to massively enhance clathrate formation, with reports of near-complete conversion to clathrate within minutes at reduced pressures and elevated temperature.^13–19^ A milestone study demonstrated H_2_-clathrates forming within an activated carbon host with a ∼ 30% reduction in pressure, 100 K increase in metastability temperature at ambient pressure, and orders of magnitude faster formation kinetics^13^ compared to pure H_2_ clathrate.^11^ Moreover, enormous quantities of natural gas occur naturally as gas clathrates within porous ocean sediments,^4,20,21^ hinting at the potential for clathrate formation in porous media. Nevertheless, the precise mechanisms behind gas clathrate promotion in porous media are incompletely understood, in part because direct experimental probing of clathrate formation within the internals of porous structures is challenging.

Several mechanisms likely contribute to the enhanced formation and stability of clathrate hydrates observed in porous media. First, the high surface area of porous structures promotes mixing between water and gas by providing extensive interfacial contact. Second, these surfaces offer abundant nucleation sites, which can significantly reduce the typically long induction times associated with clathrate nucleation.^19^ Third, the porous host may serve as a mass-transport pathway,^22^ mitigating the intrinsic limitation of slow gas diffusion through the aqueous phase and enabling more efficient delivery of guest molecules to growing clathrate structures.^23–25^ Fourth, confinement within nanoscale pores can lead to enhanced gas solubility, thereby increasing local concentrations to favorable levels for clathrate nucleation.^26–28^ Finally, improved thermal conductivity through the solid matrix of the porous medium aids in dissipating the heat released during the exothermic clathrate formation, preventing self-inhibition of growth due to local temperature rise.^29–31^ Combined, these effects make porous media *nanoreactors for clathrate formation*.^19^

Promising porous hosts for clathrate formation include activated carbons,^13,14^ metal organic frameworks,^17,18,32,33^ zeolites,^15,16,34^ and other silica-based materials.^35–37^ Activated carbon stands out due to its relatively low cost, high durability, and strong performance in gas storage applications.^13,14,38–40^ Moreover, its synthesis can be tailored to control key properties such as pore size and surface chemistry,^41,42^ both of which greatly influence clathrate formation and the subsequent H_2_ storage capacities.^19,43–46^ In particular, surface wettability can be adjusted via polar functional groups, primarily oxygen-containing species such as carbonyl (C = O), hydroxyl (C–OH) and carboxyl (COOH). Oxidative treatments increase hydrophilicity, whereas reduction enhances hydrophobicity.^47^ Experimental indications suggest that, under certain conditions, the presence of surface oxygen groups can be beneficial for clathrate formation.^48^

Prior studies have investigated the effects of pore size, gas occupancy, temperature and pressure on nanoconfined clathrate formation and stability in activated carbon.^43,44^ The role of surface chemistry in governing formation kinetics and stability, however, remains insufficiently understood. Experimental studies indicate that moderate surface wettability (neither highly hydrophilic nor strongly hydrophobic) is generally favorable for formation,^19^ allowing favorable gas–water mixing as illustrated in Figure 1. However, direct comparisons across materials are complicated, because chemical modification often alters other structural features such as pore-size distributions. Isolating the effect of surface interactions on confined clathrates therefore remains a key requirement for rational materials design.

**Figure 1:**
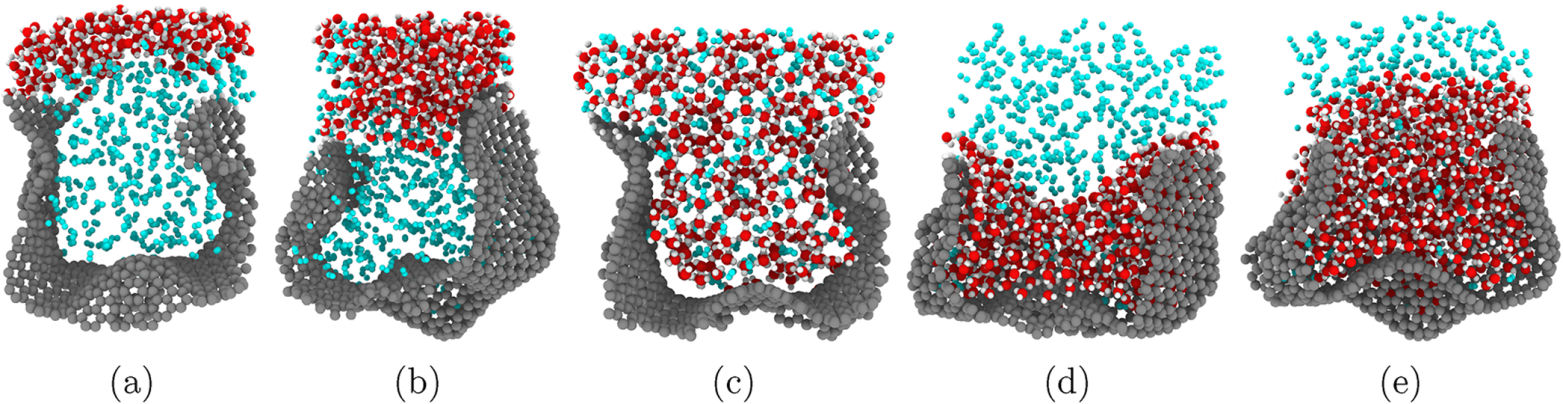
Schematic illustration of possible water and gas (H_2_) distributions in pores with varying surface wettability. From left to right: (a) a strongly hydrophobic surface, (b) a moderately hydrophobic surface, (c) an illustrative *ideal* wettability, (d) a moderately hydrophilic surface and (e) a strongly hydrophilic surface. Water molecules are shown in red and white, gas molecules are blue and the pore is represented by a gray carbon structure. Previous research suggests that strongly hydrophobic or strongly hydrophilic pores may hinder gas–water coexistence in small pores due to capillary effects, while moderate wettability, as illustrated in (b) and (d), may allow gas and water coexistance and thus be suitable for clathrate formation.^49^ Potentially, optimal wettability conditions could enable clathrate formation and stability as illustrated in (c).

This study employs molecular-dynamics simulations to determine how surface wettability influences the formation, stability and storage performance of binary H_2_–CH_4_ clathrates in activated-carbon nanopores. The binary mixture provides a high-energy-density fuel compatible with existing infrastructure and is more accessible experimentally than pure H_2_ clathrate due to lower formation pressures and faster kinetics. By varying surface interaction strength while holding pore geometry constant, the role of surface chemistry is isolated. The analysis identifies conditions that optimize formation and stability, elucidates the mechanistic basis for wettability-dependent critical pore sizes, and examines how surface interactions control dissociation kinetics. These insights support the design of porous materials that integrate clathrate-based and physisorption-based gas-storage strategies.

## 2 Methodology

### 2.1 Molecular Dynamics Simulations

All-atom molecular dynamics (MD) simulations of gas clathrate hydrate formation and dissociation within nanoporous activated carbon were performed using GROMACS 2023.3,^50^ illustrated in Figure 3. Simulations were performed in the NPT ensemble (time-step δ*t* = 2 fs) using the Nosé-Hoover thermostat^51,52^ and C-rescale barostat^53^ to capture temperature and pressure effects, with coupling constants τ_*T*_ = 0.4 ps and τ_*P*_ = 4 ps. The TIP4P/ice water model was chosen for its accurate description of phase transitions,^54^ and employed alongside OPLS-AA parametrization of CH_4_ and the activated carbon framework. The activated carbon backbone uses standard and transferrable OPLS-AA aromatic bonded and nonbonded parameters, as commonly applied to graphene, carbon nanotubes and porous carbon models, while hydroxyl surface groups adopt phenol-like parameters. H_2_ gas was modeled using a modified version of the three-point potential originally proposed by Alavi et al.^55^ This set of empirical potentials has previously been validated against *ab initio* MD using the unmodified Alavi potential.^43^ To improve agreement with *ab initio* results, particularly for configurations with multiply occupied clathrate cages which were previously found absent from H_2_-clathrate simulations,^44^ minor adjustments to H_2_-potential parameters (σ_H_2__, ϵ_H_2__, and *q*_H_2__) were applied (5–15% parameter size adjustment). The loading potential energy of different cage occupancies are shown to better match *ab initio* MD in Supporting Figure S1, and the resulting parameters are summarized in Table 1 alongside the water and activated carbon parameters. Details of the fitting procedure are provided in Supporting Section 1.

**Table 1:** Force Field Parameter Overview Including Mass (*m*), Charge (*q*), and Lennard-Jones Radius σ and Energy Well Depth ϵ^a^

**molecule**	**atom**	***m*** [**amu**]	***Q*** [**e**]	**σ** [**nm**]	**ϵ** [**kJ**/**mol**]
**H**_**2**_ **O**	O	15.9994	0	0.31668	0.88211
	H	1.008	0.5897	0	0
	Vir	0	–1.1794	0	0
**H**_**2**_	H	1.008	0.4932	0	0
	Vir	0	–0.9864	0.3038	0.2852
**CH**_**4**_	C	12.011	–0.240	0.350	0.276144
	H	1.008	0.060	0.250	0.12552
**activated carbon** *(**COH group)**	C	12.011	0	0.355	0.29288
	H	1.008	0	0.242	0.12552
	C*	12.011	0.150	0.355	0.29288
	O*	15.9994	–0.585	0.307	0.71128
	H*	1.008	0.435	0	0

a *Vir* denotes virtual sites at the center of mass of the corresponding molecules. The displayed set of activated carbon parameters correspond to ϵ_0_.

### 2.2 Fullerene-like Models of Activated Carbon

A wide range of atomistic models has been proposed for disordered and nongraphitizing carbons. Among these, the Harris fullerene–like fragment approach represents activated carbon as an assembly of curved graphene-like sheets containing pentagonal and heptagonal defects. Extensive structural characterization indicates that such fullerene-related motifs capture the curvature, disorder and pore connectivity observed experimentally in microporous carbons.^56,57^ Building on this structural framework, Terzyk and co-workers generated microporous carbons from fullerene-like fragments and used grand canonical Monte Carlo simulations to evaluate standard adsorption characterization methods. Their model reproduced realistic densities, pore size distributions and argon adsorption behavior, and were later applied to the adsorption of phenol and CF_4_, capturing expected trends with pore size and surface oxygenation.^58–61^ Another set of studies by Huang and co-workers again replicated the porosity and elemental composition of an experimental carbon using a fullerene-like model, and applied it to study benzene adsorption.^62,63^

Within the same modeling family, Di Biase and Sarkisov constructed an atomistic representation of the commercial activated carbon Maxsorb MSC-30 by packing curved, hydroxyl-functionalized fragments to match its experimentally measured surface area, micropore volume and oxygen content. They used experimental CO_2_ and CH_4_ adsorption data to tune and validate this model and showed that it reproduces single-component CO_2_ and CH_4_ isotherms at 298 K and remains predictive for these and other light gases over a range of temperatures and pressures.^64^ In a follow-up study, the calibrated Maxsorb model was used to investigate gas and gas–water mixtures relevant for pre- and postcombustion carbon capture, including multicomponent streams of CO_2_, N_2_, O_2_ H_2_, CH_4_, and H_2_O.^65^ They reported gas selectivities and influence of water content on gas adsorption in line with typical values for activated carbon. A third work focused on water sorption and scanning desorption in Maxsorb and showed that the same model captures key features of the adsorption and desorption isotherms and the associated hysteresis loop.^66^

Together, these studies establish the fullerene-fragment approach as a versatile and realistic basis for describing the pore structure and adsorption behavior of disordered carbons. In the present work, this modeling framework is used not to reproduce a specific experimental carbon but as a controlled design space in which pore size and surface interaction strength can be varied independently. This allows the influence of these key descriptors on confined H_2_–CH_4_ clathrates to be examined systematically, while remaining consistent with established structural and adsorptive characteristics of activated carbons.

### 2.3 Activated Carbon Models Used in This Work

Activated carbon microstructures were generated using Harris’ fullerene-fragment model,^56,57^ in which activated carbon consists of aggregates of curved graphene sheets. An example is shown in Figure 2a. Curvature arises from pentagonal and heptagonal defects in the otherwise hexagonal lattice structure. The hierarchical pore network generated by this method is naturally interconnected and exhibits stable percolating pathways that enable water and gas to flow between cavities, consistent with experimental observations. The activated carbon model is flexible and unconstrained, allowing mechanical relaxation and vibrational response of the surface in response to surrounding molecules. Bonded interaction parameters are listed in Supporting Table S1, and exhaustive details on activated carbon modeling are provided in a prior study.^43^ All simulation systems are treated with periodic boundary conditions, using PME electrostatics and a 0.85 nm cutoff for nonbonded van der Waals interactions, following the TIP4P/ice parametrization. Square simulation boxes of approximately (10 nm)^3^ are used encompassing around ∼1.3 × 10^5^ atoms. A previous work established the pore size as the main descriptor of the activated carbon model geometry.^43^ Structural parameters like density, fragment size, fragment curvature, etc., mainly act through their influence on the pore size distribution and have negligible direct impact on the clathrate behavior beyond their effect on porosity.

**Figure 2:**
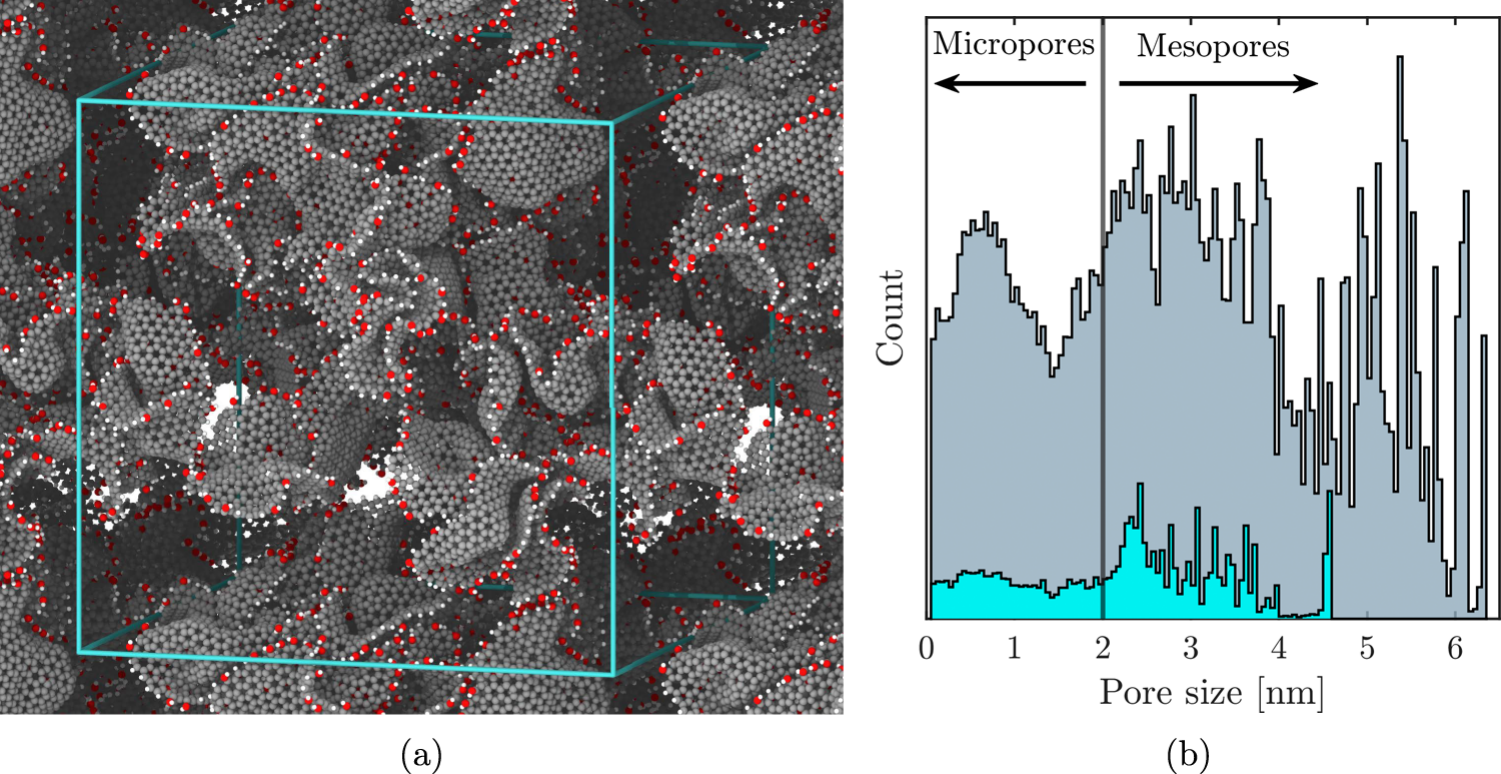
Illustration of the used activated carbon models. (a) shows a rendering of a representative activated carbon model system. The model carbon consists of curved graphene sheets in gray, with white H and red OH termination groups. The simulation system is periodically replicated in a two-dimensional plane to illustrate the porosity that an atom inside the activated carbon experiences due to periodic boundary conditions. The pore size distribution of the activated carbon in (a) is shown in blue in (b). The aggregated pore size distribution from all 10 randomized activated carbon models used in this study is shown in gray, demonstrating the range of porosity investigated.

### 2.4 Simulation Setups

This study considers three distinct cases as shown in Figure 3 equilibrium sampling of clathrate inside nanoporous activated carbon, dissociation of crystalline clathrate structures, and formation of binary clathrates from a water/gas mixture. Equilibrium sampling involved embedding a preconstructed sI clathrate crystal (with H_2_ in 80% of 5^12^-cages and CH_4_ in 6^2^5^12^-cages and remaining 5^12^-cages, yielding a molecular proportion of 20% H_2_ and 80% CH_4_) into an activated carbon model, and equilibrating at 240 K for 100 ns. Dissociation was simulated by subsequently heating the equilibrium structure to 310 at 0.5 K/ns. Melting temperatures were estimated from peaks in the dissociation rates. Formation simulations instead began with random insertion of water and gas molecules (80% CH_4_, 20% H_2_) into a nanoporous activated carbon, followed by 1.5 μs MD simulation at 260 K and 2500 bar. These conditions were previously identified as reliable for clathrate formation,^44^ with the high pressure chosen to achieve clathrate formation on simulation time scales also for the less favorable surface-interaction strengths. A binary CH_4_–H_2_ mix is used due to the difficulty of pure H_2_-clathrate formation, both experimentally and in simulations. To accelerate formation, gas molecules were incrementally added (10% increase per 100 ns) to the nonclathrate water-phase. This approach enhances the gas transport processes and induces rapid clathrate formation, but of an amorphous, kinetically arrested clathrate.^44^ The results are used for comparative analysis between different surface-interaction strengths. Clathrate content and cage structures were quantified using the Chill+^67^ and GRADE^68^ algorithms.

**Figure 3:**
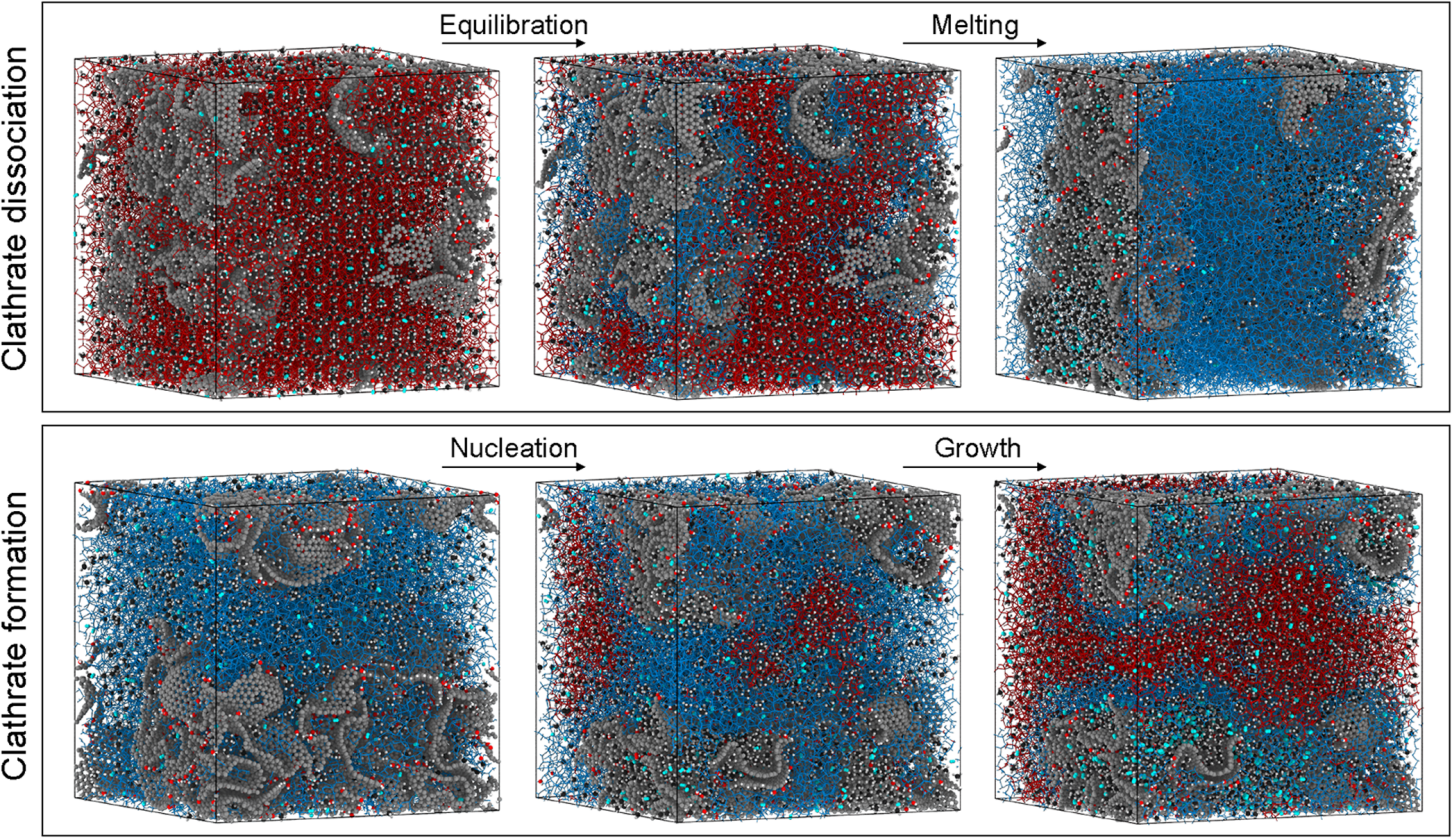
Simulation workflows. Upper panel: dissociation of gas clathrate hydrate (in red) into water (blue) and gas (light blue H_2_ and black/white CH_4_). Lower panel: formation of gas clathrate hydrate from a water–gas mixture. Dissociation has three steps: (1) initial insertion of crystalline clathrate into the activated carbon host, (2) equilibration at a temperature below melting conditions (here 220 K), and (3) gradual heating til the clathrate hydrate fully melts. Formation also has three steps: (1) initial random insertion of water and gas molecules into the activated carbon host, (2) clathrate nucleation and (3) clathrate growth.

To enhance statistical sampling, five randomized activated carbon models with varying pore sizes were employed for each case, each spanning a wide pore size distribution (Figure 2b). The pore size at any point was determined by an optimization procedure for finding the largest sphere containing that point without intersecting the porous host, as illustrated in Supporting Figure S2. Collectively, these models capture clathrate behavior in micropores and small mesopores, where surface interactions are most influential. The same models were used across all surface-chemistry variations to ensure fair comparison. However, different dynamics in different systems may lead to minor changes in the activated carbon pore structure during the simulation. Formation simulations are generally conducted in porous hosts with larger porosity than during decomposition, which is necessary to ensure consistent clathrate formation.^44^ Although this study focuses on binary H_2_–CH_4_ clathrates in activated carbon, the wide range of wettabilities and pore sizes investigated makes the findings transferable to other porous media and clathrate systems.

### 2.5 Parameterizing Surface Wettability

Nonbonded interactions between two neutral atoms *i* and *j* separated by *r⃗*_*ij*_ are modeled using the Lennard-Jones potential:

1 $$V_{\mathrm{LJ}}\left({\mathrm{r⃗}}_{\mathrm{ij}}\right)=4\epsilon_{\mathrm{ij}}\left({\left(\frac{\sigma_{\mathrm{ij}}}{{\mathrm{r⃗}}_{\mathrm{ij}}}\right)}^{12}-{\left(\frac{\sigma_{\mathrm{ij}}}{{\mathrm{r⃗}}_{\mathrm{ij}}}\right)}^{6}\right)$$

where σ_*ij*_ represents the effective atomic diameter, and ϵ_*ij*_ is the interaction strength. Interactions between different atom types use the geometric combination rules:

2 $$\sigma_{\mathrm{ij}}=\sqrt{\sigma_{i}\sigma_{j}},\epsilon_{\mathrm{ij}}=\sqrt{\epsilon_{i}\epsilon_{j}}$$

This formulation enables direct tuning of interactions between the solid surface and the surrounding species, including both liquid water and gas molecules, by adjusting only ϵ_surface_ ≡ ϵ. Interactions among water and gas molecules remain unchanged. The OPLS-AA force field is used for activated carbon, with the reference surface interaction strength ϵ_0_ = 0.29288 kJ/mol for carbon atoms. The terminal sites of carbon-sheets additionally contain H atoms or OH-groups, whose interaction strengths are modified in the same proportion as the carbon ϵ. Note that this kind of homogeneous potential modification differs somewhat from changing the surface chemistry of activated carbon via introducing or removing oxygen-containing terminal groups, which gives a less homogeneous surface interaction strength. Regardless, both approaches allow controlling surface–water interactions to investigate the effect of wettability. Furthermore, the ϵ-based approach makes the results more transferable to other porous media.

Although the ϵ parameter defines surface interaction strength in computer simulations, the preferred physical measure of surface–water affinity is typically the *wettability*, which describes how a liquid spreads on or adheres to a solid surface.^69^ Wettability is commonly quantified by the experimentally accessible contact angle θ_*C*_, defined as the angle formed between a liquid droplet and the solid surface at the three-phase boundary (solid–liquid–gas). The contact angle is governed by the balance of interfacial tensions γ through the Young equation:

3 $$\cos\left(\theta_{C}\right)=\frac{\gamma_{\mathrm{sg}}-\gamma_{\mathrm{sl}}}{\gamma_{\mathrm{lg}}}$$

where *s*, *l*, and *g* refer to solid, liquid, and gas, respectively. Strong surface interactions correspond to a hydrophilic surface (θ_*C*_ ∈ {0,90}), while weak interactions indicate hydrophobicity (θ_*C*_ ∈ {90, 180}). The extremes of the spectrum are often termed superhydrophilic or superhydrophobic.

Conveniently, prior work has established a mapping between ϵ from eq 1 and θ_*C*_ from eq 3 for graphene sheets with the same reference ϵ_0_ used here.^70^ As shown in Figure 4, this relation enables direct comparison between the simulation parameter ϵ and the experimentally measurable wettability θ_*C*_. The default activated carbon (ϵ = ϵ_0_) corresponds to a neutral wettability with θ_*C*_ ≈ 78°. At ϵ = 0.1 × ϵ_0_, the material is highly hydrophobic with θ_*C*_ ≈ 120°. Conversely, increasing ϵ to 2–3 × ϵ_0_ yields a hydrophilic surface (θ_*C*_ ∼ 43–22°), and the surface is superhydrophilic at ϵ = 10 × ϵ_0_ with an extrapolated θ_*C*_ < 1°. While the AC model exhibits considerable surface texture, the flat-sheet results of Figure 4 are adopted for simplicity and consistency, as the overall trend in wettability is more important than the precise θ_*C*_ values at each ϵ.

**Figure 4:**
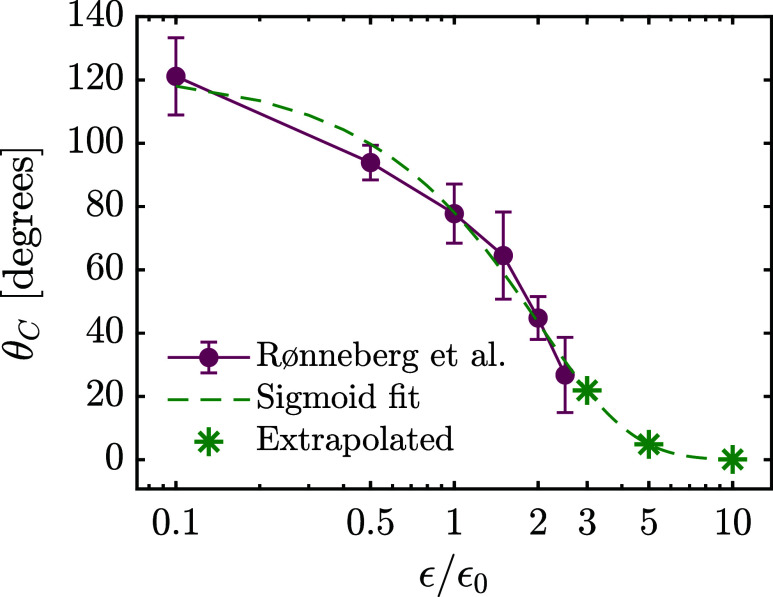
Relationship between water contact angle and the surface interaction strength ϵ. Plot generated using data reported by Rønneberg et al.^70^ and extended by sigmoid fitting to cover the range of ϵ used in this work. Error bars correspond to the standard deviation between 20 individual simulations per value of ϵ/ϵ_0_ with varying geometry.

### 2.6 Pore-Induced Melting Temperature Depression

The melting temperature *T*_*m*_ of confined clathrate hydrates is known to depend on the nanopore size via the Gibbs–Thomson effect.^9,44,71,72^ Specifically, the melting temperature depression Δ*T*_*m*_ for a clathrate in a pore of diameter *D* due to surface-effects is

4 ΔTm(D)=Tmbulk−Tm(D)=Tmbulkkgv0SΔhf,0·(γpore‐liquid−γpore‐clathrate)D=KGT(γ)D

where *T*_*m*_^bulk^ is the bulk melting temperature, *k*_*g*_ is a geometric parameter (e.g., 4 for a spherical interface in a cylindrical pore), Δ*h*_*f*,0_ is the bulk molar enthalpy of fusion, and *v*_0_^*S*^ is the molar volume of the solid clathrate hydrate. For simplicity, the strength of the melting point depression is defined as the proportionality constant *K*_GT_(γ), which theoretically could be affected by changing ϵ via the interfacial tension γ_pore–liquid_ and γ_pore–clathrate_, referring to the pore–liquid and pore–clathrate interfaces, respectively.

## 3 Results

### 3.1 Equilibrium Sampling

Increasing surface interaction strength reduces the equilibrium clathrate content by disrupting the lattice near the pore wall, as shown in Figure 5a. At high ϵ, strong interfacial interactions reorder water at the surface to maximize surface–water contact, which hinders the tetrahedral coordination required for clathrate or ice formation.^19^ This reordering propagates into the adjacent aqueous region, extending surface-induced disruption. As ϵ decreases, the total clathrate content plateaus near ϵ = ϵ_0_, indicating that hydrophobicity does not induce structural disruption beyond moderate wettability. In the limit ϵ → 0, the surface approaches noninteracting behavior and exerts negligible influence on the clathrate.

**Figure 5:**
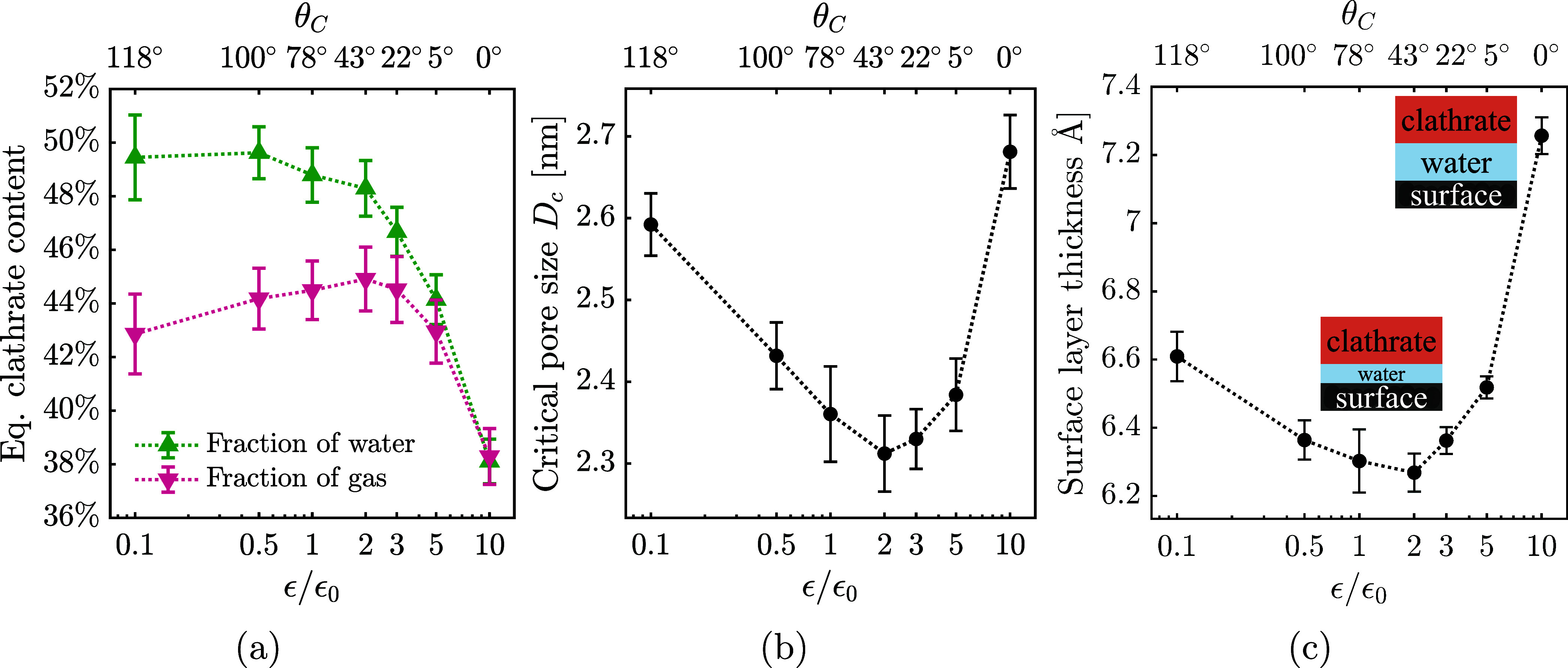
Influence of surface interaction strength ϵ on surface–clathrate equilibrium properties: (a) clathrate content, (b) critical pore size *D*_*c*_, and (c) thickness of the amorphous liquid-like premelt layer of water between the crystalline clathrate and the solid surface. The horizontal axis ϵ/ϵ_0_ is logarithmic, with error bars indicating the standard error of the mean across five repetitions of each simulation in different randomized activated carbon structures. An upper *x*-axis shows the water contact angle θ_*c*_ corresponding to each ϵ, from Figure 4. Panel (a) reports clathrate content both as the fraction of water molecules in clathrate-like structures and as the fraction of gas molecules enclathrated. Absolute clathrate content values is system-dependent, and thus serve here only for direct comparison between equivalent systems.

The total enclathrated gas content (pink curve in Figure 5a) decreases for both superhydrophilic and superhydrophobic surfaces. Strongly hydrophilic surfaces disrupt the water-ordering necessary for stable clathrate cages, directly lowering the amount of enclathrated gas. Strongly hydrophobic surfaces instead promote gas–water phase separation and generate empty cages at the clathrate–surface interface. Supporting Figure S3 shows that empty cages account for about 10% of 5^12^-cages at ϵ = 0.1 × ϵ_0_. The lower fraction of enclathrated gas compared to clathrate yield as fraction water molecules (pink versus blue curve in Figure 5a) indicates significant numbers of empty or incomplete cages that are identified structurally as clathrate but contain no gas molecules. The combined effects of structural disruption at high ϵ and phase separation at low ϵ yield a maximum in enclathrated gas content at intermediate wettability, around ϵ = 2 × ϵ_0_ (θ_*C*_ ≈ 43°).

Surface interaction strength also governs the smallest pore-diameter capable of hosting gas clathrates, referred to as the critical pore size *D*_*c*_.^43^ Defined as the smallest pore where at least 50% of water forms gas clathrate, *D*_*c*_ was previously found to be largely independent of cage structure and gas occupancy, but highly sensitive to temperature,^44^ consistent with eq 4. As shown in Figure 5b, *D*_*c*_ also depends on the surface interaction strength ϵ. At 240 K, the smallest critical pore size of 2.3 nm is observed for the moderately hydrophilic surface with ϵ = 2 × ϵ_0_, indicating maximum clathrate stability under these conditions. At high ϵ, strongly hydrophilic surfaces disrupt clathrate-like water ordering and instead favor liquid-like water within pores, thereby increasing *D*_*c*_. This disruption is further supported by Figure 5c, which shows that the amorphous water layer between the clathrate and the surface (often referred to as the intermediate layer (IML), quasi-liquid layer (QLL^73^)) and/or surface premelt layer^74^ becomes thicker with increasing hydrophilicity, indicating greater structural interference and reducing the clathrate-accessible pore volume. This layer, physically originating from the lack of tetrahedral symmetry at the surface, is studied in more detail in previous research.^74^ At low ϵ, hydrophobic pores instead promote gas accumulation with water exclusion from narrow pores, also resulting in increased *D*_*c*_. Only moderately wetting surfaces simultaneously accommodate both gas and water phases within small pores, enabling gas clathrates. This result supports the proposed influence of surface wettability on gas and water distributions within the porous network shown in Figure 1.

The disruption caused by hydrophilic surfaces in Figure 5 is examined in Figure 6. Increasing hydrophilicity produces a denser, more structured hydration layer (Figure 6a) and a narrower surface–O–H angle distribution (Figure 6b), indicating increased orientational order. Distinct peaks in the angle distribution further suggest preferred orientations that balance hydrogen bonding among water molecules with surface–water interactions. For highly hydrophilic surfaces these peaks shift to higher angles, consistent with hydrogens pointing away from the surface. At low wettability the dominant peaks occur at lower angles, with hydrogens oriented toward the surface. The fraction of hydrogen-bonded neighboring water molecules decreases with increasing ϵ (Figure 6c), evidence that the hydrogen-bond network is disrupted. Collectively, these results show that hydrophilic surfaces stabilize a more ordered yet less hydrogen-bonded interfacial layer, which disrupts the clathrate structure, broadens the intermediate water layer, increases the critical pore size, and lowers overall clathrate content.

**Figure 6:**
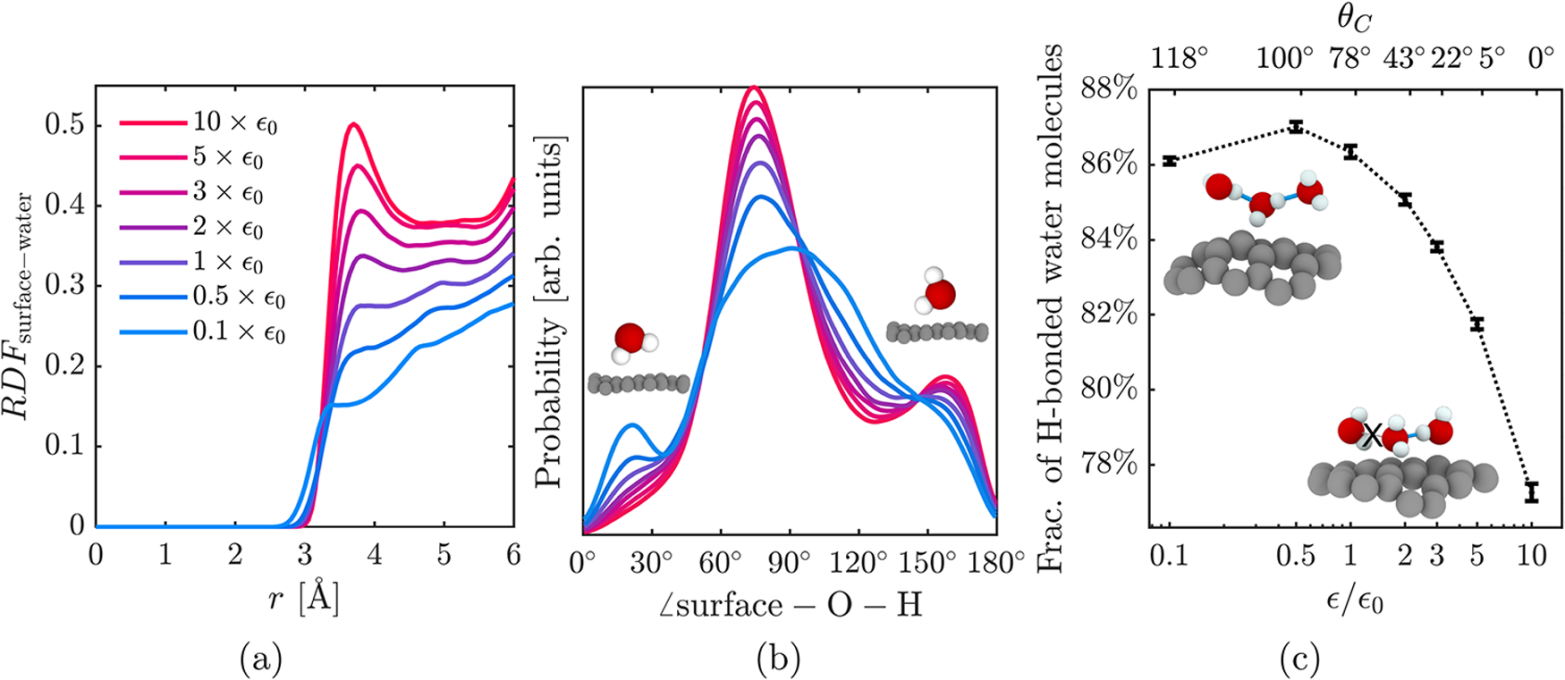
Effect of surface interaction strength ϵ on the hydration layer of water adjacent to the surface. The following properties are analyzed: (a) Radial distribution function (RDF) between surface carbon atoms and water oxygen atoms. (b) Distribution of surface–O–H angles for interfacial water molecules. Angles <90° indicate hydrogen atoms oriented toward the surface, while angles >90° indicate orientation away from the surface. (c) Fraction of neighboring water molecules (within 3.5 Å) connected by hydrogen bonds.

Surface chemistry also influences the geometry of the interfacial water layer. Supporting Figure S4 indicates that some water molecules form direct hydrogen bonds with terminal hydroxyl groups on the carbon surface, enforcing strong orientational ordering. Charged groups produce pronounced local structuring that is distinct from the homogeneous ϵ-modification applied here, which does not introduce specific binding sites. Precise control over surface–water hydrogen bonding could enable surfaces that template clathrate structure and promote nucleation and stability through enhanced interfacial ordering.^19^

### 3.2 Clathrate Dissociation

Clathrate dissociation was analyzed to quantify the effects of pore size and surface tension on the melting temperature. The results in Figure 7 show a distinct pore-size dependence, with smaller pores consistently causing earlier dissociation at all surface interaction strengths, consistent with the Gibbs–Thomson effect (eq 4). Furthermore, observed melting temperatures greatly varied with changes in ϵ. However, although variations in ϵ are theoretically expected to affect the surface-tension term *K*_GT_(γ_pore–liquid_ – γ_pore–clathrate_) in eq 4, the fitted values of *K*_GT_(γ) show variation falling within statistical error across all tested ϵ. While theory and some experimental evidence predict enhanced melting-point depression at increased surface tension (decreased wettability),^75^ the observed minimal impact aligns with earlier reports that the effect is small.^71,76^ Consequently, surface tension alone inadequately explains the observed changes in melting temperature depicted in Figure 7.

**Figure 7:**
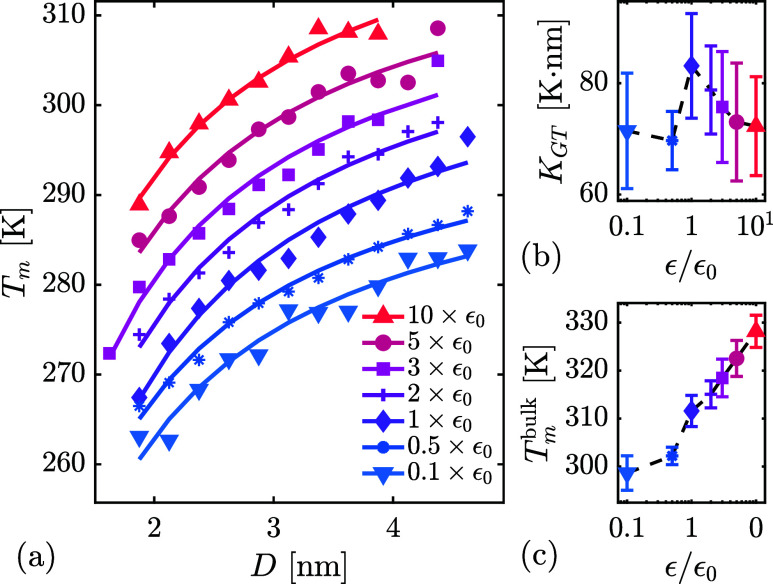
Effect of pore size and surface ϵ on clathrate dissociation. Panel (a) shows melting temperatures extracted from the peak dissociation rates (full data sets in Supporting Figure S5), grouped into bins of width 0.25 nm. Solid lines represent least-squares fits using the Gibbs–Thomson equation. Fitting parameters are displayed in panels (b) and (c) with error bars representing 95% confidence intervals. Here, $K_{\mathrm{GT}}=\frac{T_{m}^{\mathrm{bulk}}k_{g}v_{0}^{S}}{\Delta h_{f,0}}\cdot \left(\gamma_{\mathrm{pore}‐\mathrm{liquid}}-\gamma_{\mathrm{pore}‐\mathrm{clathrate}}\right)$ is the strength of the melting point depression and *T*_*m*_^bulk^ is the predicted bulk melting temperature.

Because the heating rate is finite, kinetic effects influence the observed *T*_*m*_. The reported values thus reflect a convolution of the equilibrium melting point and dissociation rate. This explains why the apparent bulk melting temperature *T*_*m*_^bulk^ varies with ϵ, even though the true bulk value is, by definition, independent of surface interactions. The melting temperature dependence on wettability thus indicates a clear interfacial kinetic effect: increased wettability slows dissociation, whereas decreased wettability accelerates it. These observations align with prior theoretical and experimental evidence that mass-transfer limitation at the dissociation interface is a dominant kinetic bottleneck.^77^ Specifically, a high local gas concentration at the interface reduces the thermodynamic driving force for decomposition.

The present simulations support a previously proposed two-step clathrate dissociation mechanism,^79,80^ in which gas first diffuses from the clathrate into the surrounding medium, followed by the collapse of empty and partially empty cages at the clathrate–water interface. The process, illustrated in Figure 8, repeats until dissociation. Under hydrophobic conditions, gas is transported efficiently out of the aqueous phase and into the porous host, lowering the interfacial gas concentration and facilitating continued dissociation (see Supporting Figure S6). Evidence includes the appearance of empty cages near the interface, low dissolved gas levels in the surrounding water phase, and a quick response to heating. In contrast, gas does not readily enter hydrophilic pores, which are effectively gas-phobic. Instead, gas must overcome a dissolution energy barrier and slowly diffuse through the water phase. This kinetic barrier limits mass transport and leads to the accumulation of dissolved gas near the clathrate–water interface. The resulting high local gas concentration creates a steep chemical potential barrier, impeding gas release from the clathrate and slowing further dissociation. Overall, hydrophilic surfaces restrict gas removal and suppress dissociation, whereas hydrophobic surfaces enhance gas transport and promote rapid breakdown, as reflected by the lower observed melting temperatures.

**Figure 8:**
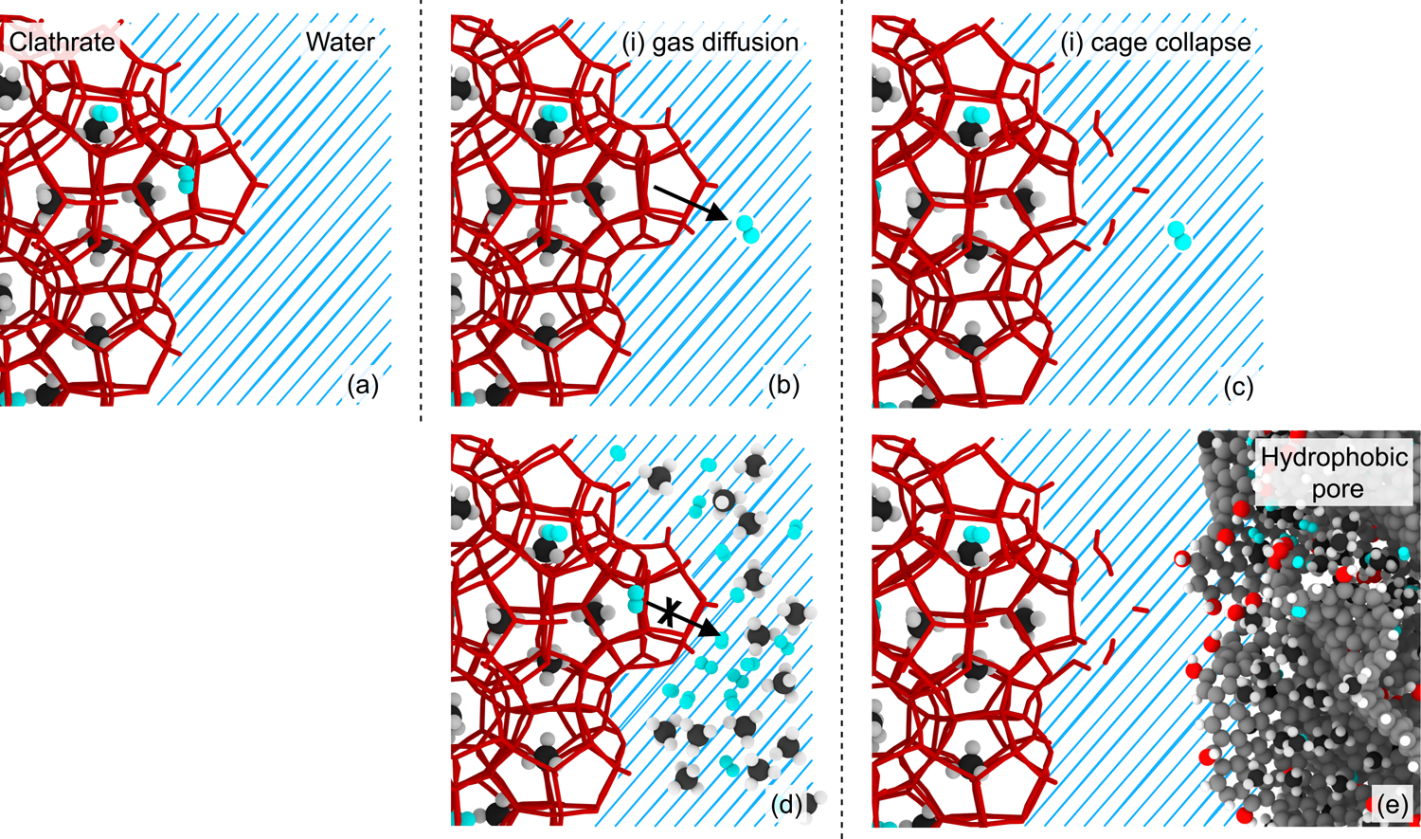
Proposed two-step clathrate dissociation mechanism. Panel (a) shows an idealized clathrate-water interface. For the dissociation front to advance, two steps are required: (i) gas diffusion out of the clathrate, illustrated in (b), and (ii) collapse of the emptied clathrate framework, illustrated in (c). An H_2_ molecule (light blue) is shown passing directly through a cage face, a pathway especially relevant for H_2_ in large 6^4^ 5^12^ cages.^78^ Gas escape at the interface can be aided by partially broken cages, temporarily disrupted hydrogen bonds between water molecules, or other defects. Panel (d) shows that the interfacial region becomes supersaturated with gas released from the dissociating clathrate, which hinders step (i). Near a hydrophobic surface, as in (e), gas is transported away more efficiently from the front, accelerating dissociation.

### 3.3 Clathrate Formation

Moderate wettability provides the most favorable conditions for clathrate formation. Figure 9a quantifies the formation dynamics across surface interaction strengths, while Supporting Figure S9 illustrates the trend qualitatively. Hydrophilic surfaces exhibit rapid nucleation and initial growth, but slow down earlier for a lower final clathrate yield. In contrast, hydrophobic surfaces initially inhibit nucleation, but growth accelerates once the local gas concentration becomes sufficiently high. Surfaces with moderate hydrophilicity (ϵ = 2–3 × ϵ_0_) consistently outperformed both extremes by balancing fast initial formation with sustained growth. The advantage of moderate wettability is further confirmed by the results in Figure 9b, which shows the smallest pore size capable of hosting clathrate formation *D*_*c*_^formation^ at different ϵ. As for equilibrium stability in Figure 5b, the minimum critical size for formation occurs at ϵ = 2 × ϵ_0_. Consequently, porous media with moderately hydrophilic surfaces maximize the volume available for clathrate formation, enhancing gas storage potential. The shown unfavorability of highly hydrophilic or highly hydrophobic surfaces, and the observation that porous media with with intermediate wettability are ideal *nanoreactors* for gas clathrate formation, is consistent with the results from prior studies.^19^ Experimental studies on methane hydrate formation in hydrophilic and hydrophobic mesoporous silicas also suggest a modest advantage for hydrophilic hosts, where synchrotron powder X-ray diffraction showed that bulk ice in the hydrophilic material is rapidly and fully converted to methane clathrate hydrate, while in the hydrophobic counterpart inactive bulk ice coexisted with the hydrate at 6 MPa.^46^

**Figure 9:**
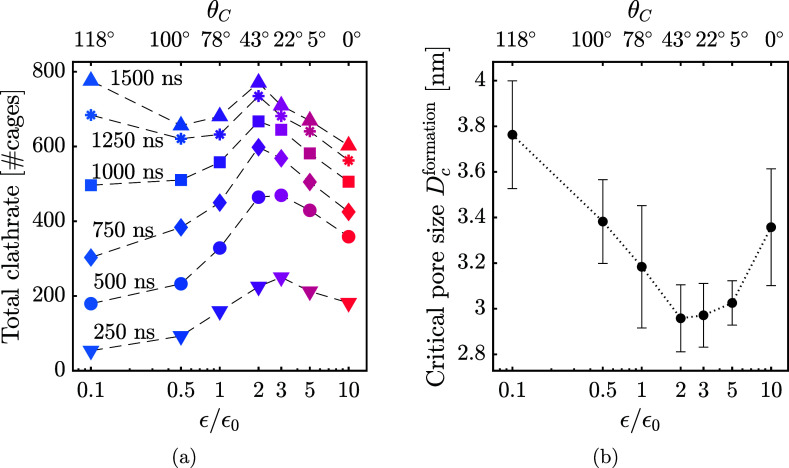
Effect of surface interaction strength on clathrate formation. (a) effect on formation rate. Total clathrate content after specific formation time is shown, averaged over the same 5 activated carbon models for each ϵ. The same data set plotted versus time for each value of ϵ/ϵ_0_ is shown in Supporting Figure S7 for an alternative visualization. (b) effect on critical pore size of formation, estimating the smallest pores in which clathrates were able to form. Extracting the critical pore size of clathrate formation involved fitting clathrate formation data to a sigmoid function, as detailed in Supporting Figure S8.

The mechanism underlying moderate wettability being preferential for clathrate formation mirrors that governing clathrate stability (previously shown in Figures 5 and 6). Strongly hydrophilic surfaces induce pronounced structuring of interfacial water, maximizing surface–water contact but inhibiting the geometric arrangement needed for clathrate cages. In contrast, highly hydrophobic surfaces encourage gas–water phase separation in small pores by preferentially adsorbing gas molecules into the carbon matrix, lowering local gas availability in the aqueous phase and hindering clathrate nucleation in small pores. Both extremes suppress the gas–water mixing required for clathrate formation, resulting in the formation critical pore size observed in Figure 9b. Notably, formation requires pores slightly larger than the minimum size that supports structurally stable clathrates (Figures 9b versus [Fig fig5]b). This formation–dissociation hysteresis implies wider stability conditions than formation conditions and has operational consequences for reversible gas loading and unloading in practical hydrogen storage applications.^43^

The clathrate cage configuration is largely insensitive to surface wettability. Clathrate structures at each ϵ are shown for equilibrium simulations in Supporting Figure S10, alongside the final structures from formation simulations in Supporting Figure S11. Both datasets show no significant differences in cage structure or occupancy across ϵ, beyond the previously noted empty cages during clathrate dissociation at ϵ < ϵ_0_. These results show that the surface wettability does not influence the clathrate structure, such as promoting sII over sI clathrates. Instead, changes in cage configuration requires alternate approaches, such as the use of thermodynamic promoter molecules.

Surface interaction strength also shapes the distribution of gas within the porous host. Physisorbed molecules are identified as those outside any water-based phase and grouped by the local pore size to obtain the distributions in Figure 10a. Low wettability (low ϵ) increases physisorption of both gases. H_2_ preferentially occupies micropores to a greater extent than CH_4_ under strongly hydrophobic conditions, whereas CH_4_ adsorbs slightly more at high wettability. Across surface chemistries, physisorption predominantly occurs in micropores below approximately 1.5 nm under the conditions studied. This observation is complemented by equilibrium simulation data presented in Figure 10b, illustrating the significant role physisorption plays in the total gas storage within a porous host with pore sizes in the 0–5 nm range. The preferential filling of the smallest pores by physisorbed gas is consistent with earlier adsorption studies in fullerene-like activated carbons, where virtual porous carbon and Maxsorb models reproduce characteristic micropore-filling behavior and identify narrow micropores as the main contributors to gas uptake.^60,64^

**Figure 10:**
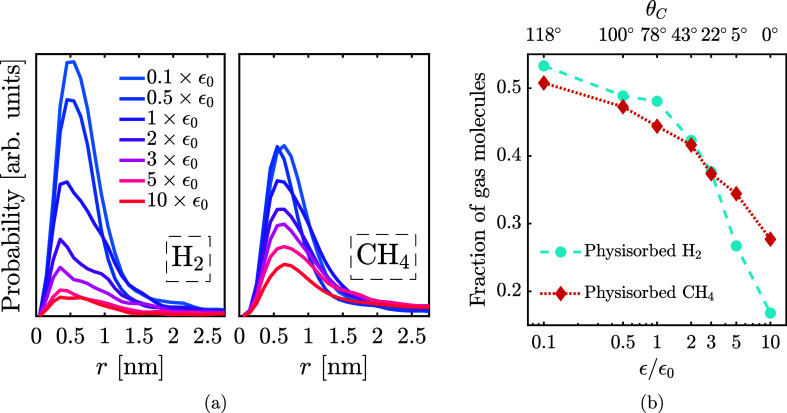
Distribution of gas molecules across the porous structure, for each surface interaction strength. (a) shows the distribution (amount of gas molecules found in pores of each size) of physisorbed H_2_ and CH_4_ in the activated carbon microporosity after 1.5 μs, shown on the same scale for comparison. (b) Fraction of each gas species that is physisorbed relative to its total number of molecules in the system. The remaining molecules of each species are either enclathrated (see Figure 5a) or dissolved in the aqueous phase (premelt layer), as shown in Supporting Figure S12. The data are taken from equilibrium simulations in which clathrate occupies all accessible pore volume at 240 K and 100 bar. Values depend on the geometry and porosity of the host and are intended for comparison across systems with equivalent structural parameters.

The distinct pore-size requirements for physisorption (micropores ≲1.5 nm) versus gas clathrates (meso- and macropores ≳2.3–3.0 nm) create opportunities to leverage a dual-storage mechanism combining both storage methods within hierarchical porous materials.^43,44^ Figure 10 highlights this synergy, showing that although physisorption favors strongly hydrophobic surfaces, moderately hydrophilic surfaces (ϵ = 2 × ϵ_0_) still maintain substantial physisorbed gas fractions, making the dual-storage mechanism viable across a broad range of surface chemistries. Materials with low to moderate wettability thus offer the best prospects for enhanced H_2_ storage via the combined mechanism. The present results complement earlier adsorption studies on fullerene-like activated carbons, which focused on optimizing gas uptake in micropores,^64–66^ by showing how additional meso- and macroporosity can be exploited for clathrate-based storage in the same class of porous hosts.

## 4 Conclusion

This work systematically investigated how surface wettability of nanoporous activated carbon hosts governs the formation, stability, and storage capacity of binary H_2_–CH_4_ clathrates. By isolating the effect of surface wettability via molecular dynamics, we identified an optimum: moderately hydrophilic surfaces (ϵ ≈ 2 × ϵ_0_, corresponding to θ_*c*_ ≈ 43°) simultaneously maximize clathrate formation rates, stability, and gas storage. This optimum arises from a balance between interfacial water structuring and gas–water phase separation. These results align with recent discussions of porous-host *nanoreactors*, where confinement and interfacial properties jointly tune hydrate nucleation and growth. Our pore-size and wettability sweep provides a direct molecular-scale realization of this principle. This supports the broader view that hydrate-promoting behavior can be engineered by tailoring nanoconfinement.

A key insight is the existence of a minimum critical pore size required for both clathrate formation and stability, which is minimized at moderate wettability. At high surface hydrophilicity, disruption of the water hydrogen-bond network leads to thick interfacial premelt layers that suppress formation in smaller pores. Conversely, strongly hydrophobic surfaces promote gas–water phase separation, destabilizing the clathrate via empty cages near the interface. Moderately wetting pores strike a balance between these effects, maximizing clathrate-accessible pore volume and enabling enclathration in smaller pores. Pores capable of hosting clathrate formation were typically 0.7–1.2 nm larger than those sustaining stable clathrates at equilibrium, with the smallest critical sizes (*D*_*c*_^formation^ ≈ 3.0 nm, *D*_*c*_ ≈ 2.3 nm) observed at moderate wettability (ϵ = 2 × ϵ_0_).

These findings also support a dual gas-storage mechanism in hierarchical porous hosts, where micropores enable physisorption and meso/macropores above the critical size support enclathration. While physisorption is favored in hydrophobic environments, moderately hydrophilic pores retain significant adsorption capacity, allowing both storage modes to coexist across a wide range of surface chemistries. This mechanism enables micropores to contribute meaningfully to gas storage capacity, despite gas calthrates themselves being inhibited at those length scales due to melting point depression, destabilizing surface–water interfacial effects, and structural instability as the crystal approaches the size of a single unit cell.

Together, the results provide clear material design guidelines for optimizing porous media for hydrogen and methane storage: combine a large volume of microporosity (<1.5 nm) for physisorption with widely developed mesoporosity above the critical size (>2.5 nm) for enclathration, and target moderate wettability (θ_*c*_ ≈ 43°) to maximize clathrate promotion while maintaining high physisorptive storage performance. Given that surface chemistry and porosity can be tailored during synthesis of activated carbon and other porous hosts, the results offer actionable guidelines for engineering porous carbon and related materials for energy storage applications.

Future work should examine the role of specific surface functional groups, including charged sites, in templating clathrate nucleation. Extension to other porous frameworks such as MOFs and zeolites will help generalize the design rules. Further investigation of porous hosts’ influence on mass and heat transport is needed, particularly through larger-scale studies of transport pathways and thermal gradients. Experimental validation remains crucial to confirm the dual-storage mechanism and to inform the synthesis of advanced porous materials for gas storage applications. Finally, dedicated studies of the adsorption behavior of this activated carbon model, including both single component and multicomponent equilibria, would further elucidate its gas storage performance and complement the clathrate focused analysis presented here.

## Supplementary Material



## Data Availability

The data underlying this study are openly available in DataverseNO at 10.18710/OGAIAG.

## Notes

Additional methodological details and result figures (PDF)
