# Supplementary material for: Optimizing Surface Wettability for Confined H2–CH4 Clathrates in Porous Activated Carbon
Source: ACS Appl Mater Interfaces. 2026 Jan 14;18(3):6098–110. doi: 10.1021/acsami.5c18795 (PMC12862775; doi:10.1021/acsami.5c18795)
Supplement: Supplementary file 1 [file am5c18795_si_001.pdf]

Supporting Information

Optimizing surface wettability for confined H_2–CH_4 clathrates in porous activated carbon

Erling Velten Rothmund, Jianying He, Zhiliang Zhang, Senbo Xiao

Department of Structural Engineering, Norwegian University of Science and Technology (NTNU), Trondheim, 7491, Norway. Corresponding author: senbo.xiao@ntnu.no

An optimized H_2 potential

The 3-point Alavi *et al.* H_2-model [1] was modified to better match *ab initio* molecular dynamics (AIMD) results of *loading potential energy*, yielding a reparametrized H_2-potential model. The loading potential energy ΔE, defined as the difference in potential energy between one filled clathrate unit cell and its separate components (empty cages and gas molecules), is:

$$\Delta E = E_{\mathrm{clathrate}}(N_{\mathrm{H_2}}) - E_{\mathrm{clathrate}}(0) - N_{\mathrm{H_2}} E_{\mathrm{H_2}}, \tag{1}$$

where $E_{\mathrm{clathrate}}$ is the potential energy of a full clathrate system with $N_{\mathrm{H_2}}$ H_2-molecules and $E_{\mathrm{H_2}}$ is potential energy of a free H_2-molecule. AIMD results, calculated at 240 K and 1350 bar, are taken from [2], using the slightly revised definition of ΔE from [3]. Details on AIMD and its comparison with force field MD (FFMD) are given in those works. To reduce variance, the present FFMD simulations used $4 \times 4 \times 4$ unit-cell boxes rather than $2 \times 2 \times 2$.

The aim of the optimization is to make the MD-derived loading energy match the AIMD reference in Equation 1. Previous H_2 clathrate-formation simulations rarely produced cages with multiple H_2 occupancy, although such states are commonly observed experimentally [3]. This discrepancy may reflect limitations of the employed force fields that bias against multiple occupancy. Although the employed TIP4P/ice water model [4] combined with the Alavi 3-point H_2 model reproduces AIMD reasonably well, it slightly favours triple over quadruple occupancy [2], and single or double occupancy are only marginally less favourable than triple. This may explain the absence of L[$4\times H_2$] states. We therefore reparametrized the H_2 potential to better represent multiply occupied cages while minimizing side effects, starting from the model of Alavi *et al.* and making only minor parameter changes of 5–15%. Two additional properties were monitored, the H_2 mean-squared displacement (MSD) and the lattice parameter a_0.

Optimization used the `scipy.optimize` simplex algorithm [5] to fit FFMD to AIMD (see Figure S1). Each iteration altered potential parameters, ran FFMD for occupancies S1L1–S2L4, evaluated ΔE, a_0, and H_2 MSD, and compared with AIMD benchmark values. Occupancies near the AIMD peak (S1L4) were weighted more heavily. AIMD and MD energy scales are shifted by a constant value (0 of energy scale is arbitrary anyway), fitting only the

occupancy-dependent shape.

The optimized potential in Figure S1 reproduces AIMD loading energies for multiply occupied cages much better than the original parametrization. The optimized model has slightly smaller molecules (lower σ) with slightly stronger Lennard-Jones interaction strength (higher ϵ), and slightly smaller partial charges (q) implying smaller polarity. These changes are consistent with other potential models for H_2 [6]. The effect of loading more H_2 into clathrate cages on the lattice parameter is slightly less accurate than the Alavi *et al.* H_2-model, likely due to smaller H_2 causing less lattice distortion. The H_2 MSD remains similar. Because accurate modelling of enclathration is the primary objective, the improved agreement in Δe justifies using this potential in the subsequent simulations.

Reducing σ improved high-occupancy energy fits but worsened low-occupancy fits and increased H_2 mobility beyond AIMD, favouring escape from cages. The inability to simultaneously fit both large and small occupancy highlights the known limitation of modelling H_2 as classical particles with single-body potentials. Classical force fields may be fundamentally insufficient for accurately modelling the quantum nature of light H_2 molecules in confinement. Both nuclear and electronic quantum effects are likely necessary to accurately capture the complex interactions between H_2 and the clathrate cage environment, with one investigation finding that nuclear quantum effects increase energetic barriers for H_2 leaving multiply occupied cages [7, 8]—an effect which would help stabilize multiply occupied H_2-clathrate cages.

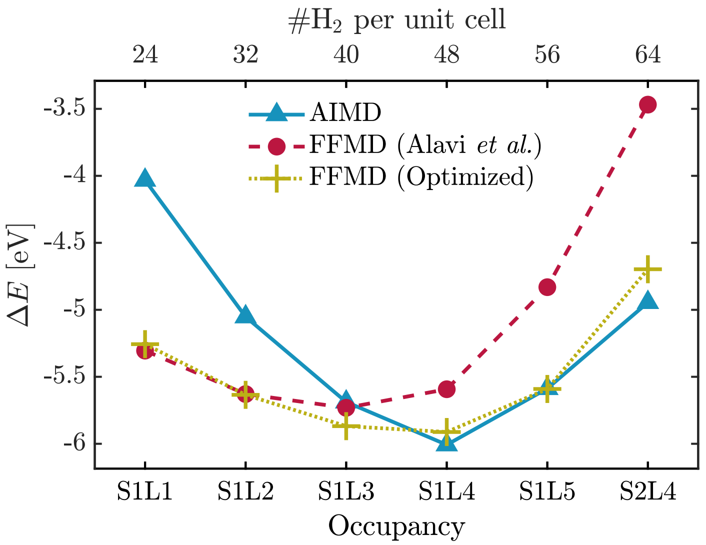

(a)

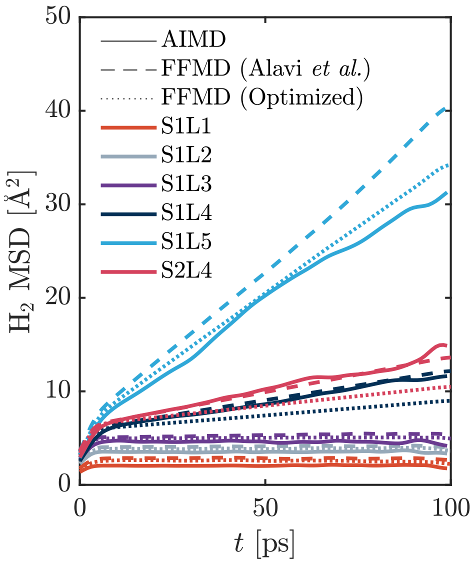

(b)

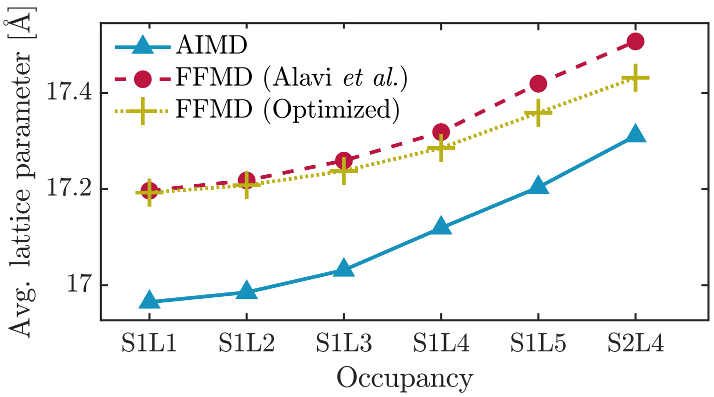

(c)

(d) H_2-potential parameters in the original and optimized 3-point hydrogen models. σ and ϵ parameters are for the center-of-mass virtual site, which has charge $-2 \times Q$. The individual H atoms have charge Q.

version	Q [e]	σ [nm]	ϵ [kJ/mol]
Alavi et al.	0.493	0.3038	0.2852
Optimized	0.443	0.2890	0.3230

Figure S1 A new parametrization for the H_2 potential. Starting from the 3-point Alavi *et al.* H_2-model, an optimization approach produced a revised H_2-potential that more closely follows results from *ab initio* molecular dynamics (AIMD). The AIMD benchmarks are taken from a previous study [2, 3]. The effect of cage-occupancy on three properties were considered, (a) loading potential energy ΔE, (b) H_2 msd (a measure of mobility), and (c) the clathrate lattice parameter. The resulting parameters are summarized in (d).

Supplementary methods

Table S1 Overview of bonded interaction parameters used in this work. Atoms marked with * belong to terminal OH groups. Bonds involving H-atoms are constrained (marked with -), i.e. kept at fixed distance b^0. Water molecules are fully rigid. See [9] for details on how interactions are calculated.

Bonds

Molecule	i	j	b^0 [nm]	k^b [kJ mol^{-1} nm^{-2}]
AC	C	C	0.14200	392459.2
	C	H	0.10800	-
	C	C*	0.15100	265265.6
	C*	O*	0.13640	376560.0
	O*	H*	0.09450	-
H_2	H	H	0.07414	-
CH_4	C	H	0.10900	-
H_2O	O	H	0.09572	-

Angles

Molecule	i	j	k	θ^0 [°]	k^θ [kJ mol^{-1} rad^{-2}]
AC	C	C	C	120.000	527.184
	C	C	H	120.000	292.880
	C	C	C*	120.000	585.760
	C	C*	C*	114.000	527.184
	C(*)	C*	O*	109.500	418.400
	C*	O*	H*	113.000	292.880
CH_4	H	C	H	107.800	276.144
H_2O	H	O	H	104.52	-

Dihedrals (Proper dihedrals: Ryckaert-Bellemans type)

Molecule	i	j	k	l	C_1	C_2	C_3	C_4	C_5	C_6	[KJ mol^{-1}]
AC	C/H	C	C	C/H	30.33400	0	−30.33400	0	0	0	
	C	C*	O*	H*	7.03749	0	−7.03749	0	0	0	

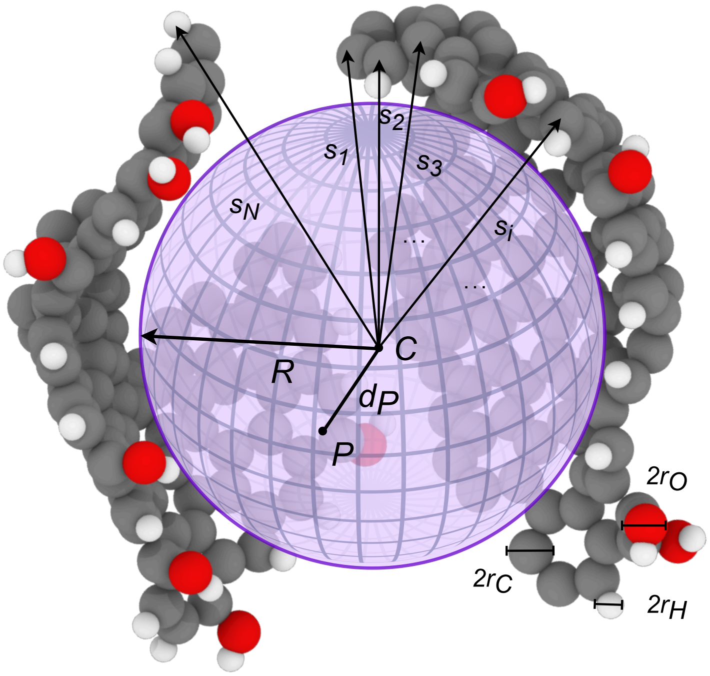

Figure S2 Pore size calculations in activated carbon. Following the constrained optimization problem proposed by Bhattacharya & Gubbins [10], the pore size at a point P is defined as the diameter $D = 2R$ of the largest sphere centred at C that contains P without intersecting any surrounding atoms. The radius $R(C)$ is determined by the minimum distance s_i from C to each atom i minus the atom's radius $r_i \in r_C, r_O, r_H$. The point P lies inside the sphere if its distance to C, denoted $d_P = |P - C|$, is smaller than the radius of the sphere $R(C)$. i.e.,

$$\begin{gathered}\text{MAXIMIZE}\{R(C)\} \text{ where } R(C) = \text{MIN}(s_i - r_i),\ i = 1, 2, ..., N \\ \text{subject to constraint} \\ R(C) - d_P \geq 0, \quad d_P = |P - C|,\end{gathered} \tag{2}$$

Supplementary results

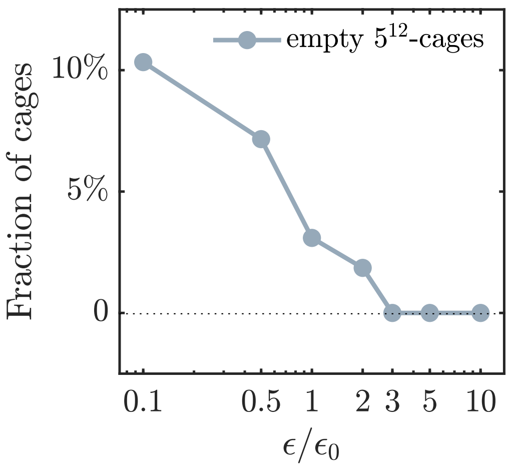

Figure S3 Fraction of small 5^{12}-cages containing no gas molecules during simulations of equilibrium clathrate crystals inside porous activated carbon with different surface interaction strength ϵ. The full cage structure and occupancy at each ϵ is shown in supplementary Figure S10.

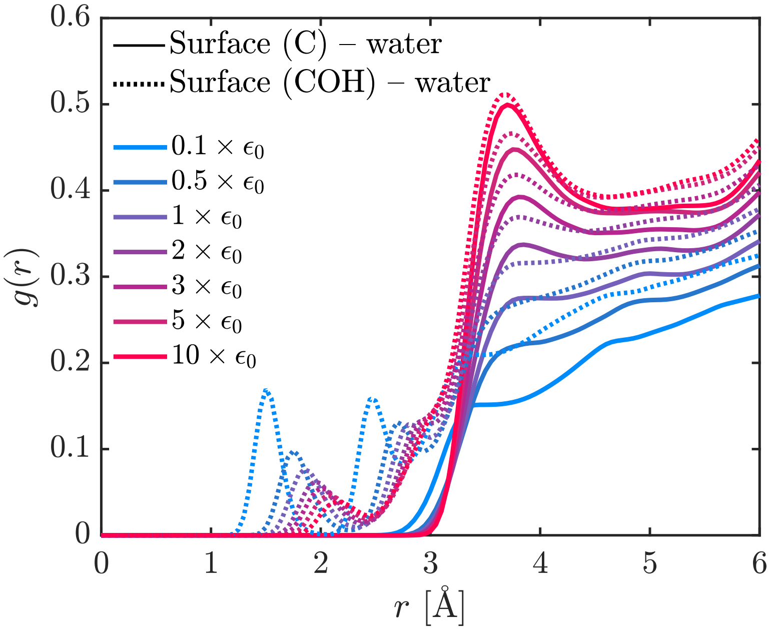

(a)

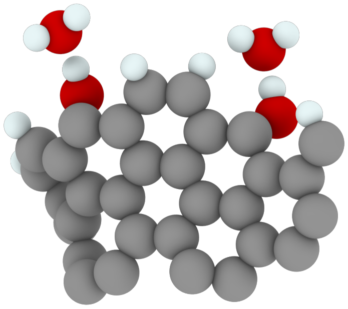

(b)

Figure S4 Hydrogen bonding between water and surface OH groups. (a) Radial distribution functions $g(r)$ between surface atoms and water oxygen-atoms. Comparing all surface atoms to carbon-only shows additional short-range peaks when OH groups are included. These peaks indicate direct hydrogen bonding at OH sites, with two peaks separated by 0.95 Å, matching O–H bond-distance of the OH groups in the used potential. (b) Rendering of two water molecules hydrogen-bonded to surface OH sites. Direct hydrogen bonding is more pronounced at low surface interaction strength ϵ, because electrostatic interactions between polar sites and water become relatively stronger when uncharged nonbonded interactions are weaker.

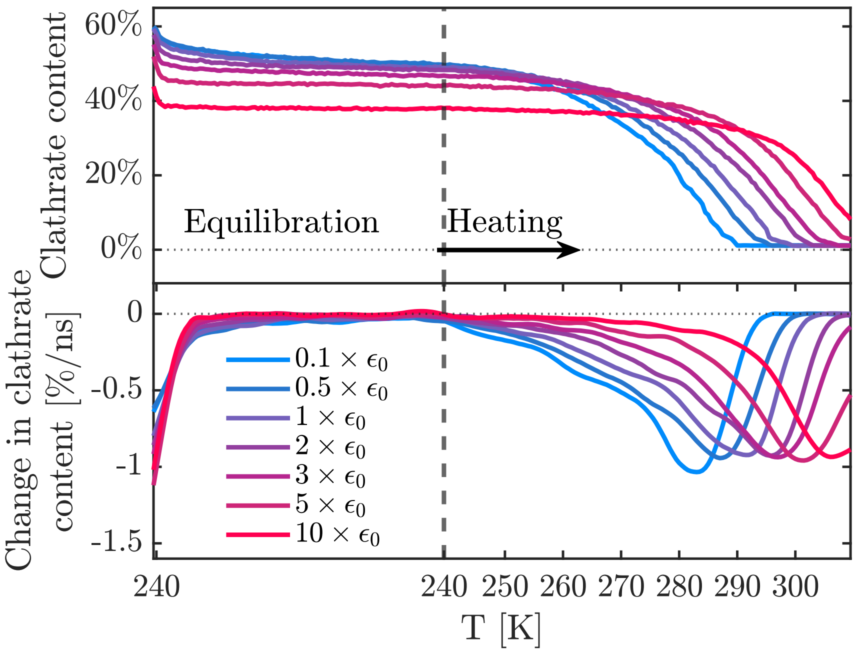

Figure S5 Melting trajectories of binary H_2–CH_4 clathrate (20/80 % mix) at 100 bar under gradually increasing temperature for different surface interaction strength ϵ. The upper panel shows clathrate content as the relative fraction of all water molecules in the system forming a clathrate phase. The lower panel shows the corresponding rate of dissociation. The temperature at the peak clathrate dissociation rate corresponds to the melting temperatures T_m. Each trajectory is the average of five independent MD simulations with different randomized activated carbon structures.

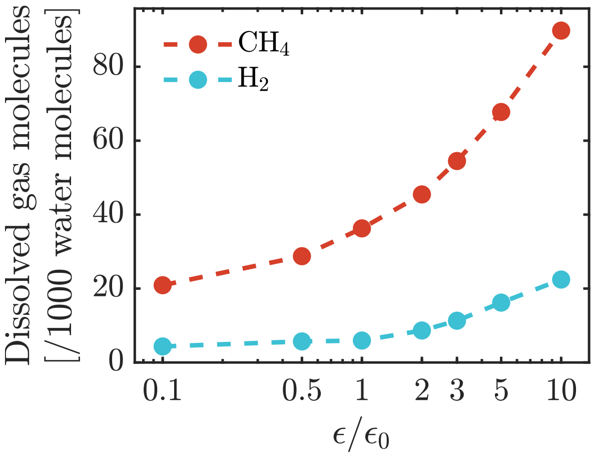

Figure S6 Molecular gas concentration in the water phase during dissociation at 240 K and 100 bar, shown for each ϵ. The overall gas composition is 80% CH_4 and 20% H_2 by number of molecules. Gas concentrations far exceed the normal solubility limits of CH_4 or H_2 in liquid water (respectively 1.4 mmol/L and 0.8 mmol/L or gas/water ratio of 2.5e-5 and 1.4e-5 for CH_4 and H_2 at atmospheric pressure and room temperature [11, 12], which is around 3 orders of magnitude lower than observed here). Higher solubility is expected here due to elevated pressure, lower temperature, confinement effects [13–17], and being in the immediate vicinity of high gas-concentration gas clathrate and microporous activated carbon saturated with gas molecules (at some ϵ). Nevertheless, the simulated concentrations remain higher than expected equilibrium values, indicating slow mass-transport of gas out of the water-phase. The effect is much more pronounced at high ϵ. Further, the closed NPT setup prevents complete removal of dissolved gas to an external sink, which must be considered when interpreting results which are sensitive to changes in gas concentration.

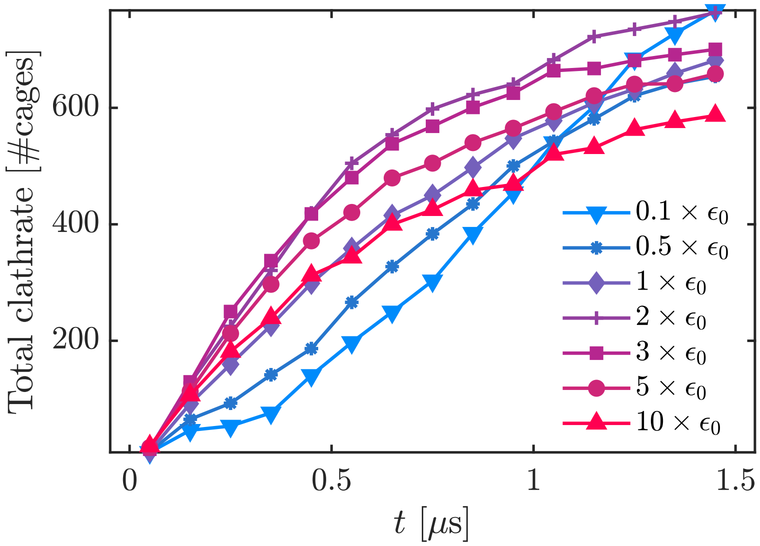

Figure S7 Effect of surface interaction strength on clathrate formation. Each curve shows the time evolution of total clathrate content, averaged over five independent simulations using different activated carbon structures. The hydrophobic surfaces exhibit delayed initial clathrate growth, likely due to the surface increasing water–gas dissociation kinetics, lowering effective gas concentration and slowing clathrate formation. However, the slow formation results in a slightly more crystalline clathrate with increased cage density (see [3]), causing an overall higher total clathrate amount, similar to the surfaces with moderate wettability. The surfaces with higher wettability triggered fast initial formation but they converge towards a lower final clathrate content.

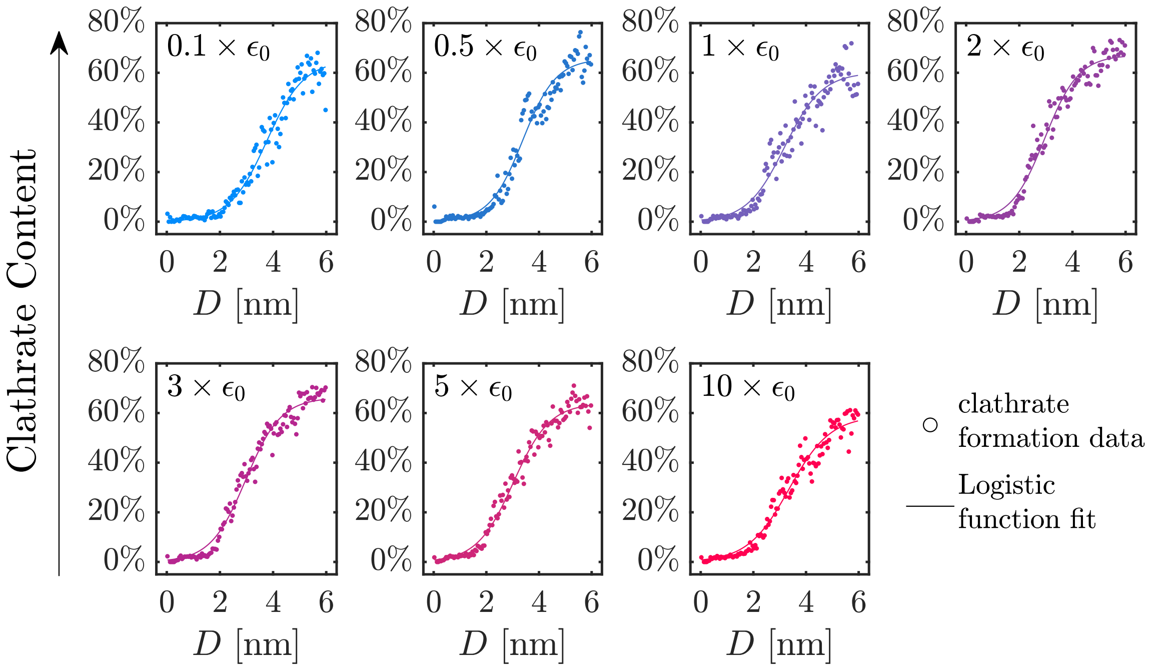

Figure S8 Determination of the critical pore size by fitting clathrate content versus pore diameter D to a logistic (sigmoid) function. Clathrate content refers to the fraction of water molecules within pores of diameter D classified as clathrate. Because the formed clathrate is highly amorphous, the total content saturates well below 100% even in bulk, which makes a fixed-threshold definition ($\geq 50\%$) unsuitable. Instead, the critical pore size of formation was determined as the midpoint of the fitted sigmoid curve (inflection point), capturing the transition from pores not allowing clathrate formation to those that do. The logistic model describes the data well and provides consistent, robust estimates of the critical pore size, with values summarized in Figure 9b of the main article.

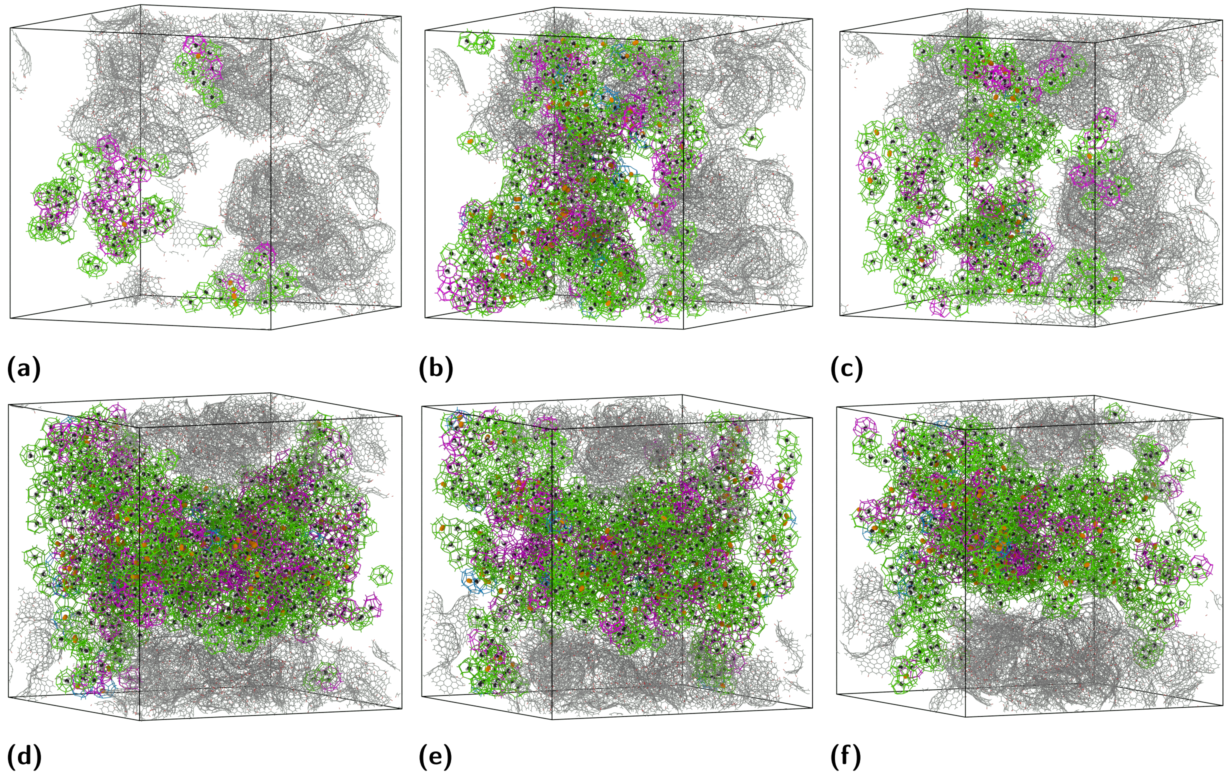

Figure S9 Rendering of clathrate cage structures in representative system snapshots during formation simulations. The clathrate structure after 500 ns growth in activated carbon is shown for (a) a superhydrophobic surface ($\epsilon = 0.1 \times \epsilon_0$), (b) a surface with moderate wettability ($\epsilon = 2 \times \epsilon_0$), and (c) a superhydrophilic surface ($\epsilon = 10 \times \epsilon_0$). Clathrate after 1.5 μs growth in another activated carbon model is shown in (d-f) with the same wettability as (a-c). For clearer visualization, only water molecules forming complete clathrate cages are shown (green 5^{12}, purple $6^2 5^{12}$, and very rare blue $6^4 5^{12}$). Enclathrated gas molecules are shown as black CH_4 and orange H_2, and the activated carbon is displayed in gray.

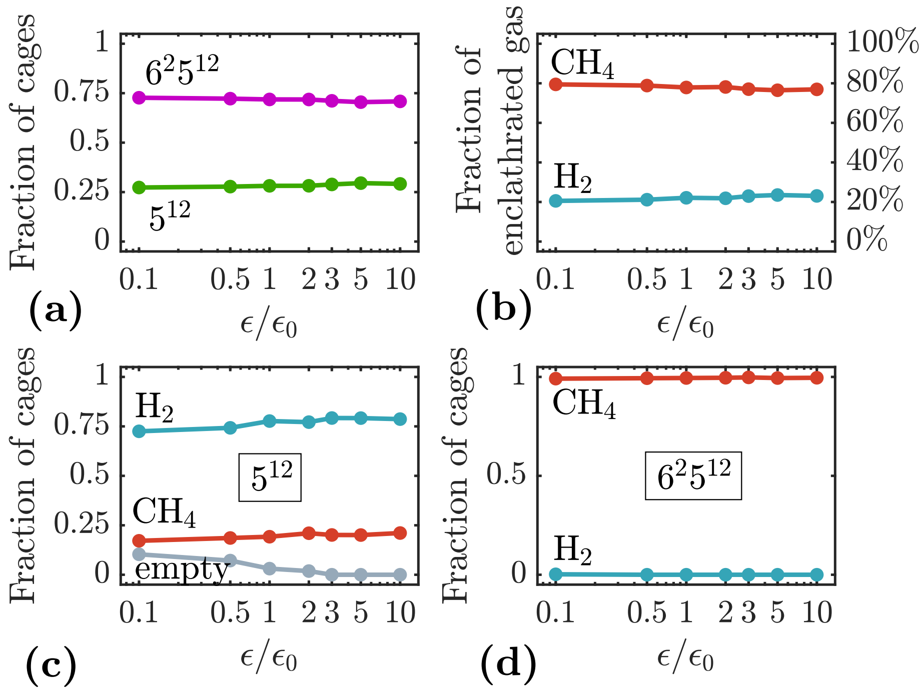

Figure S10 Average clathrate cage structure at 240 K and 100 bar as a function of surface interaction strength ϵ. Panel (a) counts the number of S- (5^{12}), M- ($6^2 5^{12}$), and L-cages ($6^4 5^{12}$). Panel (b) shows the fraction of enclathrated gas molecules of each species. Panels (c–d) show the specific cage occupancies of S- and M-cages, with no L-cages present. Notably, the surface has no marked influence on the cage-structure. The only minor influence is the presence of empty S-cages at low ϵ.

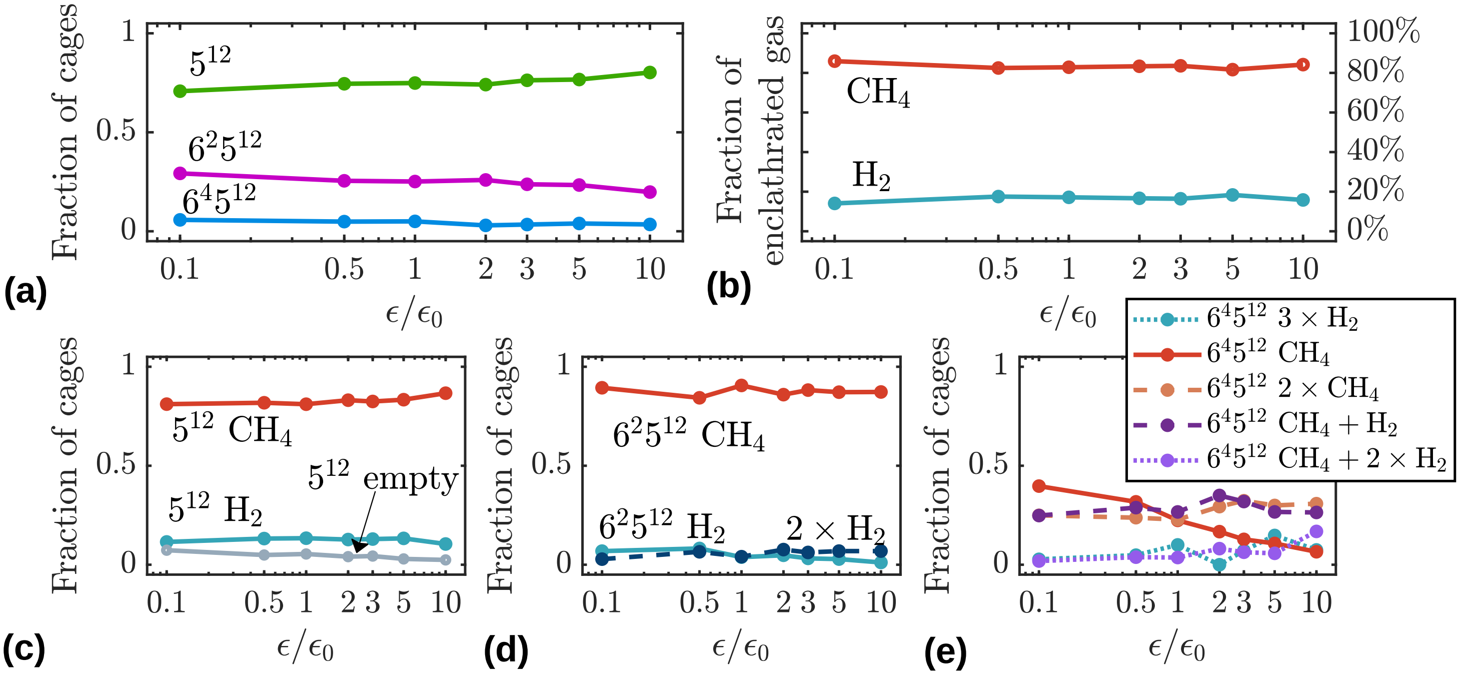

Figure S11 Average clathrate cage structure at 270 K and 100 bar as a function of surface interaction strength ϵ. The data is accumulated from five clathrate formation simulations performed in different activated carbon structures for each ϵ. Panel (a) counts the number of S- (5^{12}), M- (6^25^{12}), and L-cages (6^45^{12}). Panel (b) displays the fraction of enclathrated gas molecules of each species. Panels (c–e) show the specific cage occupancies of S-, M-, and L-cages. The slight increase in S-cages at high ϵ is explained by the increased water-phase gas saturation at these conditions (Figure S6), previously shown to favour kinetically arrested amorphous clathrates with an excess of S-cages [3]. L-cages display a myriad of different cage occupancies. At low ϵ, L-cages mainly contain a single CH_4, gradually transitioning towards multiple CH_4 and combined H_2+CH_4 occupancy at high ϵ. These L-cage occupancies are expected to vanish as the clathrate approaches its equilibrium structure. Because the total number of L cages is <small, most specific L-cage occupancies are rare and contribute negligibly to the averages.

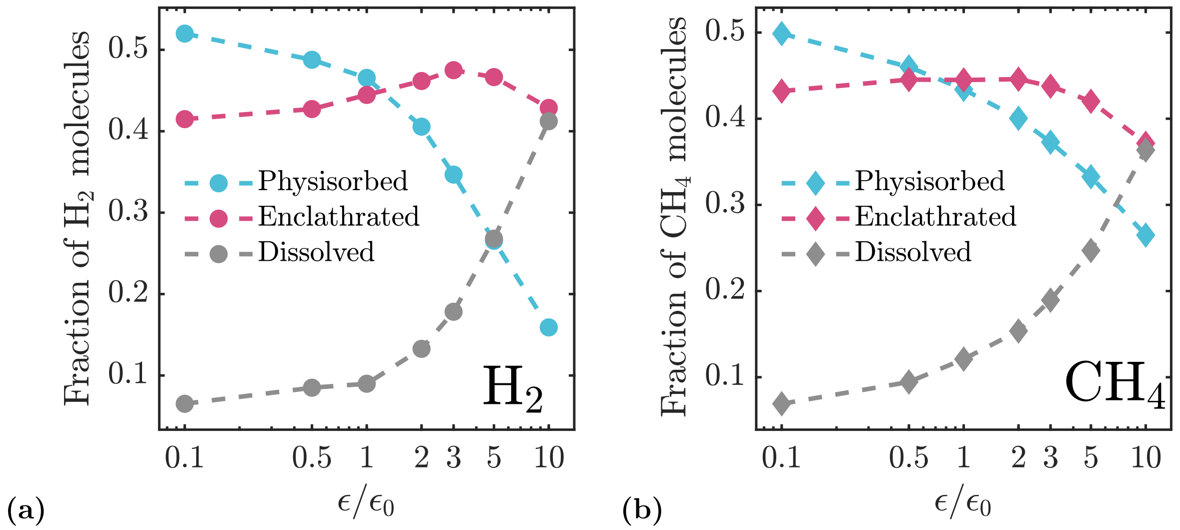

Figure S12 Fraction of (a) H_2 and (b) CH_4 molecules that are physisorbed, enclathrated, or dissolved within the intermediate liquid-like premelt layer. Enclathration trends are discussed in Figure 5a of the main article and physisorption in Figure 10b. The dissolved fractions of both gases exceed their bulk solubilities in water and would ordinarily diffuse out of the system, but this is prevented by the NVT ensemble. The data are taken from equilibrium simulations in which clathrate occupies as much of the pore space as permitted by the structure at 240 K and 100 bar. Numerical values depend on the geometry and porosity of the medium and are intended for comparison between equivalent systems.

References

[1] S. Alavi, J. A. Ripmeester and D. D. Klug, 'Molecular-dynamics study of structure II hydrogen clathrates,' *The Journal of Chemical Physics*, vol. 123, no. 2, p. 024 507, Jul. 2005, ISSN: 0021-9606. DOI: 10.1063/1.1953577. eprint: https://pubs.aip.org/aip/jcp/article-pdf/doi/10.1063/1.1953577/15368223/024507_1_online.pdf.

[2] E. V. Rothmund, J. He, Z. Zhang and S. Xiao, 'Revealing the critical pore size for hydrogen storage via simultaneous enclathration and physisorption in activated carbon,' *Journal of Materials Chemistry A*, vol. 12, no. 33, pp. 21 830–21 844, 2024, ISSN: 2050-7496. DOI: 10.1039/d4ta03607g.

[3] E. V. Rothmund, J. He, Z. Zhang and S. Xiao, 'Promoting hydrogen storage under mild conditions by binary clathrate hydrates in porous activated carbon,' *Chemical Engineering Journal*, p. 166 696, 2025, ISSN: 1385-8947. DOI: https://doi.org/10.1016/j.cej.2025.166696.

[4] J. L. F. Abascal, E. Sanz, R. García Fernández and C. Vega, 'A potential model for the study of ices and amorphous water: Tip4p/ice,' *The Journal of Chemical Physics*, vol. 122, no. 23, Jun. 2005, ISSN: 1089-7690. DOI: 10.1063/1.1931662.

[5] P. Virtanen *et al.*, 'SciPy 1.0: Fundamental Algorithms for Scientific Computing in Python,' *Nature Methods*, vol. 17, pp. 261–272, 2020. DOI: 10.1038/s41592-019-0686-2.

[6] M. Barraco, S. Neyertz, N. E. Benes and D. Brown, 'Comparison of eight classical lennard-jones-based h2 molecular models in the gas phase at temperatures and pressures relevant to hydrogen on-board storage tanks,' *The Journal of Physical Chemistry A*, vol. 127, no. 30, pp. 6335–6346, Jul. 2023, ISSN: 1520-5215. DOI: 10.1021/acs.jpca.3c03212.

[7] C. J. Burnham, Z. Futera and N. J. English, 'Quantum and classical inter-cage hopping of hydrogen molecules in clathrate hydrate: Temperature and cage-occupation effects,' *Physical Chemistry Chemical Physics*, vol. 19, no. 1, pp. 717–728, 2017, ISSN: 1463-9084. DOI: 10.1039/c6cp06531g.

[8] S. Alavi, D. D. Klug and J. A. Ripmeester, 'Simulations of structure ii h2 and d2 clathrates: Potentials incorporating quantum corrections,' *The Journal of Chemical Physics*, vol. 128, no. 6, Feb. 2008, ISSN: 1089-7690. DOI: 10.1063/1.2825618.

[9] M. Abraham *et al.*, *Gromacs 2025.2 manual*, version 2025.2, May 2025. DOI: 10.5281/zenodo.15387070.

[10] S. Bhattacharya and K. E. Gubbins, 'Fast method for computing pore size distributions of model materials,' *Langmuir*, vol. 22, no. 18, pp. 7726–7731, Aug. 2006, ISSN: 1520-5827. DOI: 10.1021/la052651k.

[11] A. Blackman, *Aylward and findlay's si chemical data*, eng, Milton, 2014.

[12] P. Englezos, S. Alavi and J. A. Ripmeester, 'Kinetics of clathrate hydrate processes,' in *Clathrate Hydrates*. John Wiley & Sons, Ltd, 2022, ch. 14, pp. 631–716, ISBN: 9783527695058. DOI: https://doi.org/10.1002/9783527695058.ch14. eprint: https://onlinelibrary.wiley.com/doi/pdf/10.1002/9783527695058.ch14.

[13] Z. Zhu, Y. Cao, Z. Zheng and D. Chen, 'An accurate model for estimating h2 solubility in pure water and aqueous nacl solutions,' *Energies*, vol. 15, no. 14, p. 5021, Jul. 2022, ISSN: 1996-1073. DOI: 10.3390/en15145021.

[14] J. Grabowska *et al.*, 'Solubility of methane in water: Some useful results for hydrate nucleation,' *The Journal of Physical Chemistry B*, vol. 126, no. 42, pp. 8553–8570, Oct. 2022, ISSN: 1520-5207. DOI: 10.1021/acs.jpcb.2c04867.

[15] B. Coasne and D. Farrusseng, 'Gas oversolubility in nanoconfined liquids: Review and perspectives for adsorbent design,' *Microporous and Mesoporous Materials*, vol. 288, p. 109 561, Nov. 2019, ISSN: 1387-1811. DOI: 10.1016/j.micromeso.2019.109561.

[16] C. Carrillo-Carrión, J. Farrando-Perez, L. L. Daemen, Y. Q. Cheng, A. J. Ramirez-Cuesta and J. Silvestre-Albero, 'Zr-porphyrin metal–organic framework as nanoreactor for boosting the formation of hydrogen clathrates,' *Angewandte Chemie International Edition*, vol. 63, no. 6, Jan. 2024, ISSN: 1521-3773. DOI: 10.1002/anie.202315280.

[17] S. Yu, R. Zheng, Q. Kang and M. Mehana, 'Predicted tenfold increase of hydrogen solubility in water under pore confinement,' *Environmental Chemistry Letters*, vol. 22, no. 3, pp. 945–951, Feb. 2024, ISSN: 1610-3661. DOI: 10.1007/s10311-024-01698-3.
